# CRISPR-mediated deletion of prostate cancer risk-associated CTCF loop anchors identifies repressive chromatin loops

**DOI:** 10.1186/s13059-018-1531-0

**Published:** 2018-10-08

**Authors:** Yu Guo, Andrew A. Perez, Dennis J. Hazelett, Gerhard A. Coetzee, Suhn Kyong Rhie, Peggy J. Farnham

**Affiliations:** 1 Department of Biochemistry and Molecular Medicine and the Norris Comprehensive Cancer Center, Keck School of Medicine, University of Southern California, 1450 Biggy Street, NRT 6503, Los Angeles, CA 90089-9601 USA; 2 Department of Biomedical Sciences and the Samuel Oschin Comprehensive Cancer Institute, Cedars-Sinai Medical Center, Los Angeles, CA 90048 USA; 3 Van Andel Research Institute, Grand Rapids, MI 49503 USA; 4 Department of Biochemistry and Molecular Medicine and the Norris Comprehensive Cancer Center, Keck School of Medicine, University of Southern California, 1450 Biggy Street, NRT G511B, Los Angeles, CA 90089-9601 USA

**Keywords:** CTCF, GWAS SNPs, CRISPR, Prostate cancer, Chromatin looping

## Abstract

**Background:**

Recent genome-wide association studies (GWAS) have identified more than 100 loci associated with increased risk of prostate cancer, most of which are in non-coding regions of the genome. Understanding the function of these non-coding risk loci is critical to elucidate the genetic susceptibility to prostate cancer.

**Results:**

We generate genome-wide regulatory element maps and performed genome-wide chromosome confirmation capture assays (in situ Hi-C) in normal and tumorigenic prostate cells. Using this information, we annotate the regulatory potential of 2,181 fine-mapped prostate cancer risk-associated SNPs and predict a set of target genes that are regulated by prostate cancer risk-related H3K27Ac-mediated loops. We next identify prostate cancer risk-associated CTCF sites involved in long-range chromatin loops. We use CRISPR-mediated deletion to remove prostate cancer risk-associated CTCF anchor regions and the CTCF anchor regions looped to the prostate cancer risk-associated CTCF sites, and we observe up to 100-fold increases in expression of genes within the loops when the prostate cancer risk-associated CTCF anchor regions are deleted.

**Conclusions:**

We identify GWAS risk loci involved in long-range loops that function to repress gene expression within chromatin loops. Our studies provide new insights into the genetic susceptibility to prostate cancer.

**Electronic supplementary material:**

The online version of this article (10.1186/s13059-018-1531-0) contains supplementary material, which is available to authorized users.

## Background

Prostate cancer (PCa) is the leading cause of new cancer cases and the third cause of cancer death among men in the USA [1]. Of note, 42% of prostate cancer susceptibility can be accounted for by genetic factors, the highest among all cancer types [2]. Therefore, it is of critical importance to understand the underlying genetic mechanisms that lead to PCa. Investigators have used genome-wide association studies (GWAS) to investigate the genetic components of risk for PCa. The first step in GWAS employs arrays of 1–5 million selected single nucleotide polymorphisms (SNPs), which allows the identification of risk-associated haplotype blocks in the human genome. Because large regions of the human genome are inherited in blocks, each risk locus potentially contains many risk-associated SNPs. Fine-mapping studies are next performed to more fully characterize these risk loci, identifying the SNPs that are in high linkage disequilibrium with the GWAS-identified index SNP and that are most highly associated with disease risk (as defined by allelic frequencies that are statistically most different between cases and controls). To date, GWAS has identified more than 100 prostate cancer risk loci [3–7], with subsequent fine-mapping studies employing both a multi-ethnic and a single large European population identifying at least 2,181 PCa risk-associated SNPs [8–10]. Although considerable progress has been made in identifying genetic variation linked to disease, the task of defining the mechanisms by which individual SNPs contribute to disease risk remains a great challenge. One reason for this lack of progress is because a great majority of the risk-related SNPs lie in non-coding regions of the genome. Thus, the GWAS field has been left with the conundrum as to how a single nucleotide change in a non-coding region might confer increased risk for a specific disease. These non-coding risk-associated SNPs clearly do not affect disease risk by changing the function of a specific protein but rather it is thought that a subset of these SNPs may contribute to changes in levels of expression of a key protein or non-coding regulatory RNA [11–15]. Deciphering which risk-associated SNP is likely to be a functional SNP (i.e., a SNP that contributes to changes in gene expression) and not simply a “hitchhiker” SNP is the first step in a post-GWAS study [12, 16]. We reasoned that risk-associated SNPs lying within regulatory elements are more likely to be causal, rather than hitchhiker SNPs. Therefore, our approach, described in detail below, was to perform a comprehensive analysis of the regulatory potential of all prostate cancer risk-associated SNPs identified by the fine-mapping studies, by comparing the location of each SNP to regulatory elements (promoters, enhancers, insulators, and chromatin loop anchors) that are active in prostate cells. Using this approach, we reduced the set of 2,181 fine-mapped PCa risk-associated SNPs to a smaller set of ~ 300 candidate functional SNPs. After selecting the subset of SNPs that are in active regulatory regions, we next assayed the effects of removal of a small genomic region harboring a SNP-containing regulatory element on gene expression [12]. Using CRISPR-mediated deletion of candidate functional PCa risk-associated SNPs at two risk loci, we have identified long-range loops that function to repress gene expression.

## Results

### Identification of PCa risk-associated regulatory elements

**Fig. 1:**
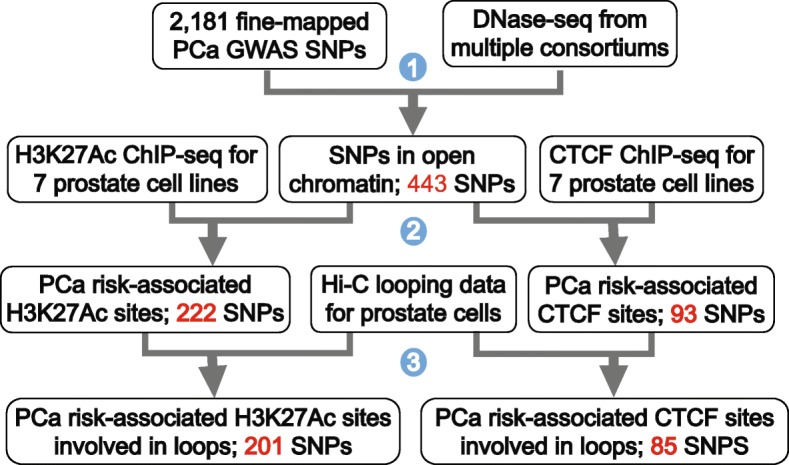
Experimental and analytical steps used to identify PCa risk-associated regulatory elements involved in chromatin loops. Step (1): The subset of 2,181 fine-mapped PCa-associated SNPs that overlap a DNase hypersensitive site was identified. Step (2): H3K27Ac and CTCF ChIP-seq was performed in duplicate in two normal (PrEC and RWPE-1) and five cancer (RWPE-2, 22Rv1, C4-2B, LNCaP, and VCaP) prostate cell lines; data was collected plus or minus DHT for 22Rv1 and LNCaP cells, for a total of 18 datasets for each mark (36 ChIP-seq samples). The SNPs in open chromatin sites (i.e., those that are contained within a DHS site) were then subdivided into those that overlap a H3K27Ac or a CTCF site in prostate cells; the number of PCa-associated SNPs associated with the H3K27Ac or CTCF sites is shown. Step (3): The PCa risk-associated H3K27Ac and CTCF sites were overlapped with Hi-C looping data, and the subset of each type of site involved in chromatin loops was identified; the number of PCa-associated SNPs associated with the H3K27Ac or CTCF sites involved in looping is shown

Our goal in this study was to identify PCa risk-associated SNPs that are important in regulating gene expression (e.g., by their influence on the activity of distal enhancers or via their involvement in maintaining 3D chromatin structure). As described above, fine-mapping has been previously performed to expand the set of prostate cancer GWAS index SNPs to a larger set of 2,181 PCa risk-associated SNPs that are potential causal variants [8–10]. As our first step (Fig. 1), we determined which of the 2,181 fine-mapped PCa SNPs are located within known DNase hypersensitive sites (DHS). We began with this comparison because, in contrast to ChIP-seq peaks of histone modifications which are fairly broad, DHS sites identify relatively narrow regions of open chromatin that closely correspond to the transcription factor (TF) binding platform of regulatory elements. By first requiring the SNPs to overlap a DHS, we reduce the number of “false-positive” SNPs that lie at the outer margins of broad ChIP-seq peaks. To capture as many SNPs within regulatory elements as possible, we obtained a set of 2.89 million DHS peaks that have been identified from a large number of human cell lines and tissues (downloaded from the ENCODE project portal at encodeproject.org). Overlapping the genomic coordinates of these DHS with the genomic locations of the set of fine-mapped PCa risk-associated SNPs identified 443 SNPs located within open chromatin.

Because we used DHS sites from more than 100 cell or tissue samples, many of the SNP-associated regulatory elements may not be active in prostate cells. Therefore, as a second step, we identified the subsets of DHS-localized SNPs that are within H3K27Ac or CTCF ChIP-seq peaks that are present in prostate cells. Studies of cultured prostate cancer cells and sequencing of prostate cancers have revealed multiple distinct subgroups of prostate cancer [17], including prostate cancer cells that are refractory to androgen treatment, that contain the androgen receptor splice variant AR-V7, or that express fusion proteins such as TMPRSS2-ERG. Because we wished to capture SNPs in regulatory elements that are present in multiple prostate cancer subgroups, as well as in normal prostate cells, we performed H3K27Ac and CTCF ChIP-seq in two non-tumorigenic prostate cell populations (PrEC and RWPE-1) and five prostate cancer cell lines (RWPE-2, 22Rv1, C4-2B, LNCaP, and VCaP). PrEC are normal human prostate epithelial primary cells whereas RWPE-1 is a normal prostate epithelial cell line that was immortalized by transfection with a single copy of human papilloma virus 18 [18]. RWPE-2 cells were derived from RWPE-1 cells by transformation with the Kirsten murine sarcoma virus [18]. LNCaP is an androgen-sensitive prostate adenocarcinoma cell line derived from a lymph node metastasis [19]. C4-2B is a castration-resistant prostate cancer cell line derived from a LNCaP xenograft that relapsed and metastasized to bone after castration [20]; C4-2B cells do not require androgen for proliferation, having similar growth rates in the presence or absence of androgen [21]. VCaP cells are derived from a metastatic lesion to a lumbar vertebrae of a Caucasian male with hormone refractory prostate cancer; VCaP is a TMPRSS2-ERG fusion-positive prostate cancer cell line, expressing high levels of the androgen receptor splice variant AR-V7 [22]. 22Rv1 is a castration-resistant human prostate carcinoma epithelial cell line that is derived from an androgen-dependent CWR22 xenograft that relapsed during androgen ablation [23]; this cell line also expresses the androgen receptor splice variant AR-V7. Unlike most prostate cancer cell lines, 22Rv1 has an almost diploid karyotype.

**Fig. 2:**
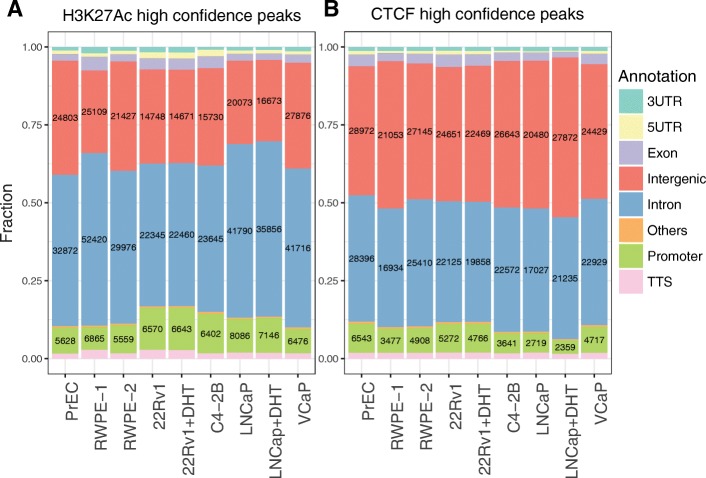
Identification and classification of H3K27Ac (**a**) and CTCF (**b**) sites in prostate cells. H3K27Ac and CTCF ChIP-seq was performed in duplicate for each cell line; for 22Rv1 and LNCaP cells, ChIP-seq was performed in duplicate in the presence or absence of DHT. Peaks were called for individual datasets using MACS2 and the ENCODE3 pipeline, then peaks present in both replicates were identified (high confidence peaks) and used for further analysis (see Additional file 3: Table S2). The location of the peaks was classified using the HOMER annotatePeaks.pl program and the Gencode V19 database. The fraction of high confidence peaks in each category is shown on the *Y* axis, with the number of peaks in each category for each individual cell line and/or treatment shown within each bar

**Fig. 3:**
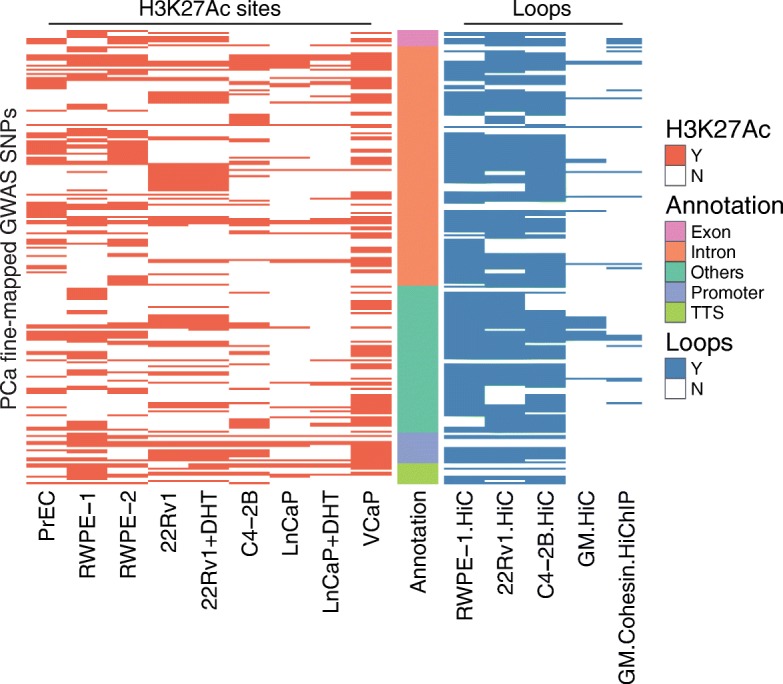
PCa risk SNPs associated with H3K27Ac sites and chromatin loops. Each row represents one of the 222 SNPs that are associated with both a DHS site and a H3K27ac peak in normal or tumor prostate cells (Additional file 4: Table S3). The location of each SNP was classified using the Gencode V19 database. “Others” represents mostly intergenic regions. To identify the subset of H3K27Ac-associated risk SNPs located in an anchor point of a loop, chromatin loops were identified using Hi-C data from normal RWPE-1 prostate cells [26] or 22Rv1 and C4-2B prostate tumor cells (Rhie et al., in preparation); Hi-C [25] and cohesin HiChIP data [27] from GM12878 was also used

**Fig. 4:**
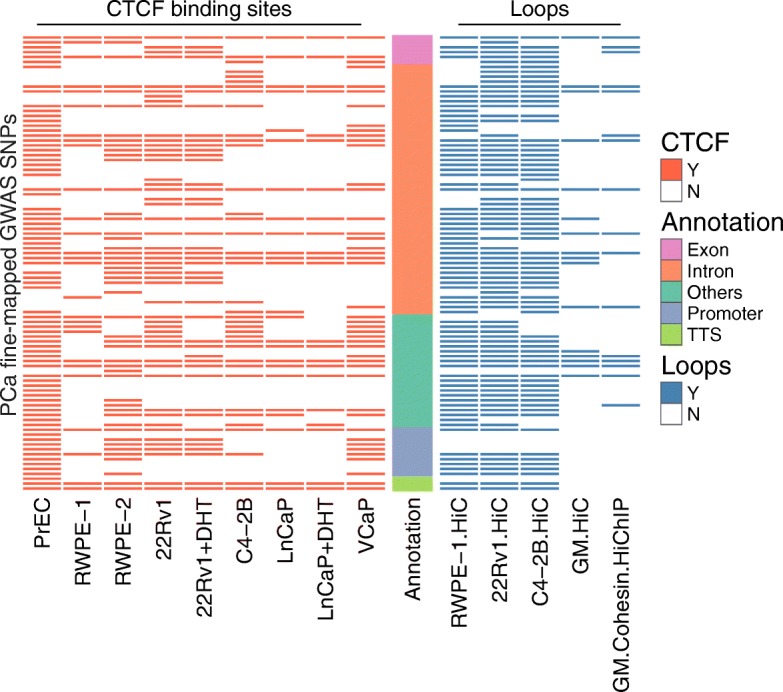
PCa risk SNPs associated with CTCF sites and chromatin loops. Each row represents one of the 93 SNPs that are associated with both a DHS site and a CTCF peak in normal or tumor prostate cells (Additional file 4: Table S3). The location of each SNP was classified using the Gencode V19 database. “Others” represents mostly intergenic regions. To identify the subset of CTCF-associated risk SNPs located in an anchor point of a loop, chromatin loops were identified using Hi-C data from normal RWPE-1 prostate cells [26] or 22Rv1 and C4-2B prostate tumor cells (Rhie et al., in preparation); Hi-C [25] and cohesin HiChIP data [27] from GM12878 was also used

Each ChIP-seq was performed in duplicate and, for 22Rv1 and LNCaP cells, in the presence or absence of dihydrotestosterone (DHT), for a total of 18 datasets for each mark (36 ChIP-seq experiments in total). Peaks were called for individual datasets using MACS2 and the ENCODE3 pipeline [24], and only high confidence (HC) peaks (defined as those peaks present in both replicates) were used for further analysis; see Additional file 1: Figure S1 for ranked peak graphs for each HC peaks dataset, Additional file 2: Table S1 for information concerning all genomic datasets created in this study, and Additional file 3: Table S2 for lists of the HC ChIP-seq peaks for H3K27Ac and CTCF for each cell line. As shown in Fig. 2, we identified 48,796–94,688 H3K27Ac and 43,157–69,945 CTCF sites that were reproducible in the two replicates from each cell line and growth condition. As expected from other studies, most of the H3K27Ac and CTCF sites were either located in introns or were intergenic, with a small subset located in promoter regions (defined as 1 kb upstream to + 100 bp downstream from a known TSS). A comparison of the set of DHS-localized SNPs to the union set of H3K27Ac or CTCF HC peaks from the prostate cells identified 222 PCa risk-associated SNPs located within a DHS site that corresponds to an H3K27Ac peak (Fig. 3) and 93 PCa risk-associated SNPs located within a DHS site that corresponds to a CTCF peak (Fig. 4).

### Using 3D chromatin interaction datasets to identify PCa risk-associated enhancer and CTCF sites involved in long-range looping

In previous studies, we found that deletion of a regulatory element that has active histone marks does not always alter the transcriptome [13]. This suggests that not all regulatory elements (even if marked by H3K27Ac) are critically involved in gene regulation in that particular cell type under those particular conditions (perhaps due to functional redundancy of regulatory elements). We reasoned that one way to identify critical regulatory elements could be to focus on the subset that is involved in chromatin looping. Although analysis of Hi-C data suggests that many of the long-range chromatin loops (e.g., those that are anchored by CTCF sites and that define topological associating chromatin domains (TADs)) are common to multiple cell types, intra-TAD loops may be cell type-specific [25]. Therefore, we performed in situ Hi-C [25] in normal prostate RWPE-1 cells [26] and in the prostate cancer cell lines C4-2B and 22Rv1 (Rhie et al., manuscript in preparation). For comparison, we also obtained Hi-C and cohesin HiChiP datasets from GM12878 cells [25, 27]. We then overlapped PCa risk-associated DHS+, K27Ac+ SNPs with the genomic coordinates of the anchors of the identified loops, identifying 203 SNPs located in the DHS portion of a H3K27Ac ChIP-seq peak and associated with a chromatin loop (Fig. 3); a list of these risk SNPs can be found in Additional file 4: Table S3. Most of these SNPs are located in intronic or intergenic regions, and many are located in loops present in both prostate and GM12878 cells. We performed similar experiments overlapping the PCa risk-associated DHS+, CTCF+ SNPs with the loop anchor regions and identified 85 SNPs located in the DHS portion of a CTCF ChIP-seq peak and associated with a chromatin loop (Fig. 4); see Additional file 4: Table S3. Again, the majority of these SNPs are located in intronic or intergenic regions.

### Functional analysis of prostate cancer risk-associated CTCF sites

**Fig. 5:**
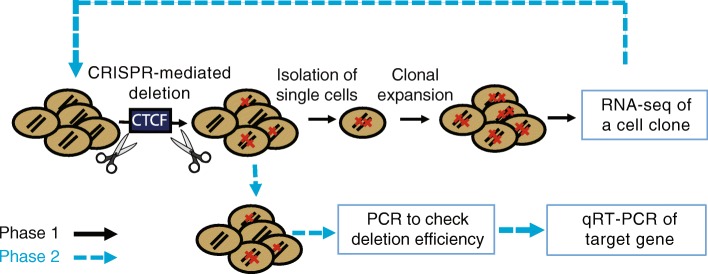
Experimental workflow for functional investigation of PCa risk-associated CTCF sites. Phase 1: Plasmids encoding guide RNAs that target sequences on each side of a PCa risk-associated CTCF site were introduced into the PCa cell line 22Rv1 along with a Cas9 expression vector (see the “Methods” section for details). The resultant cell pool was analyzed to determine deletion efficiency (red slashes represent alleles in each cell that harbor a CTCF site deletion). Single cells were then selected and expanded into clonal populations for RNA-seq analysis. Phase 2: After identifying the gene most responsive (within a ± 1-Mb window) to deletion of the region encompassing a risk-associated CTCF site, plasmids encoding guide RNAs that target the risk-associated CTCF anchor region and/or the regions encompassing the CTCF sites looped to the risk CTCF site and a Cas9 expression plasmid were introduced into 22Rv1 cells; cell pools were analyzed by PCR to check deletion frequency and by RT-qPCR to measure expression of the target gene

CTCF has been shown to affect gene regulation by several different mechanisms. For example, TADs are formed by interaction of two convergently bound CTCFs separated by a large number of base pairs (500 kb to 1 Mb) [25, 28–31]; the physical interaction of the CTCFs bound to each anchor point creates a chromatin loop. CTCF is also thought to influence enhancer-mediated gene regulation, functioning in both positive and negative ways. For example, CTCF may help to bring an enhancer closer in 3D space to a target promoter via its ability to form intra-TAD loops with other CTCF sites. In contrast, binding of CTCF at a site between an enhancer and promoter can, in some cases, block long-range regulation (see the “Discussion” section). To determine if PCa risk-associated CTCF anchor regions that we identified to be involved in looping do in fact control the expression of specific genes, we used the CRISPR/Cas9 system to delete PCa risk-associated CTCF anchor regions and then assessed the effects of these deletions on the transcriptome (Fig. 5; see also Additional file 5: Table S4 for sequences of guide RNAs used for all deletion studies). Unlike most PCa cells, 22Rv1 cells are diploid; therefore, we have used these cells for our CRISPR/Cas9 experiments. We chose to study two PCa risk-associated CTCF anchor regions, one on chr1 and one on chr12. These regions are both located in intergenic regions of the genome and thus are not easily associated a priori with a specific target gene. Also, these regions are robustly bound by CTCF in all nine HC peak sets and are identified as being involved in 3D chromatin looping in all of the Hi-C or HiChIP datasets that we analyzed. Although the chosen PCa risk-associated SNPs are not located precisely within the CTCF motif, they are within CTCF peaks. In a previous study of allele-specific differences in binding strength of CTCF in 51 lymphoblastoid cell lines, the authors found that the majority of the nucleotide changes associated with CTCF binding strength were within 1 kb of the CTCF-binding motif (or in linkage disequilibrium with a variant within 1 kb of the motif) but very few were actually in the CTCF motif itself [32].

**Fig. 6:**
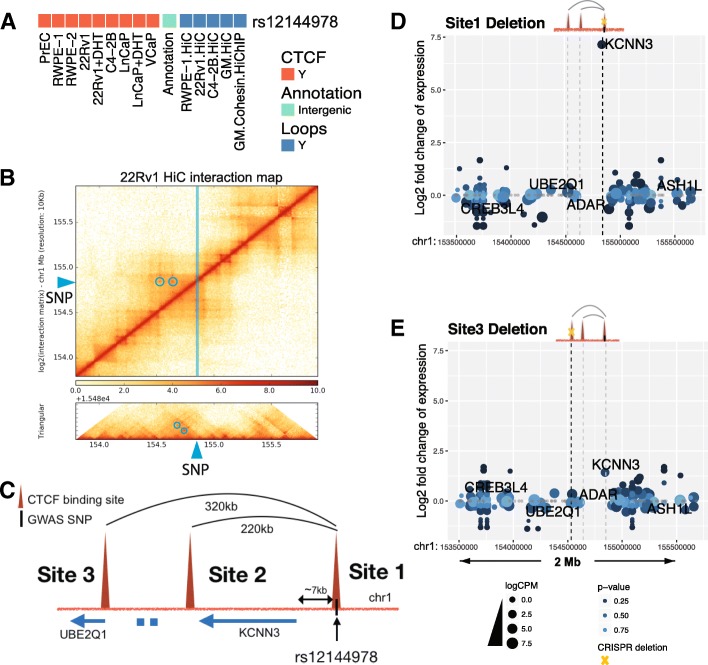
*KCNN3* is upregulated upon targeted deletion of the region encompassing the CTCF site near rs12144978. **a** A blowup of the CTCF peak information, genomic annotation, and looping information for rs12144978 from Fig. 4. **b** Hi-C chromatin interaction map of the region of chromosome 1 near the rs12144978. The location of the SNP is indicated by the blue line and arrow. The blue circles indicate the high confidence risk loops used in the analysis. **c** Detailed schematic of the high confidence risk loops in which rs12144978 is involved, as identified by the Hi-C chromatin interaction data. **d** Shown is the fold-change expression of all genes within a ± 1-Mb region near rs12144978 in the cells deleted for a 1607-bp region encompassing the PCa risk-associated CTCF site (site 1); a volcano plot illustrating the genome-wide analysis of the RNA-seq data can be found in Additional file 1: Figure S2. The yellow X indicates which CTCF site has been deleted. **e** Shown is the fold-change expression of all genes within a ± 1-Mb region near rs12144978 in the cells deleted for a 913-bp region encompassing CTCF site 3. The yellow X indicates which CTCF site has been deleted

**Fig. 7:**
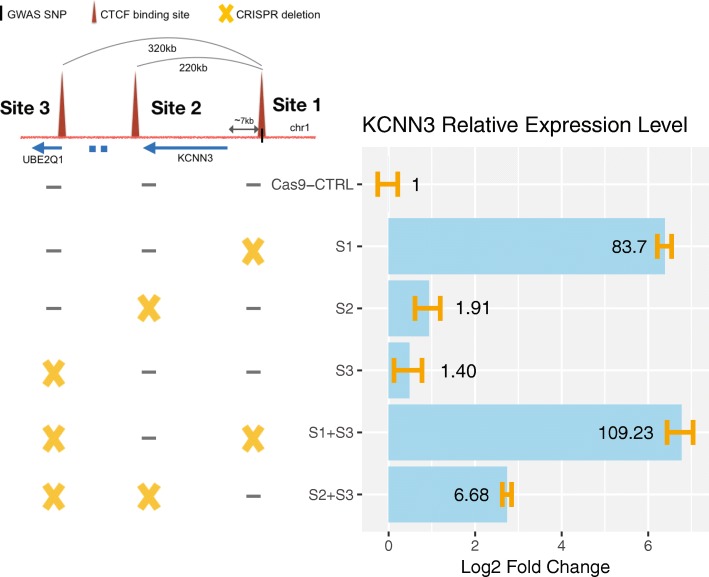
Analysis of the rs12144978-associated chromatin loops. Guide RNAs targeting regions encompassing CTCF site 1 (the PCa risk-associated CTCF site), CTCF site 2, and/or CTCF site 3 (or the empty guide RNA vector as a control) were introduced into 22Rv1 prostate cancer cells, along with Cas9. Cell pools were harvested, and *KCNN3* expression was analyzed by RT-qPCR. Shown within the blue bars is the fold change in *KCNN3* expression in the pools that received guide RNAs vs. the vector control. The yellow X indicates which CTCF site has been deleted; the size of each deletion can be found in Additional file 5: Table S4

We began by deleting the CTCF anchor region on chr1 near PCa risk-associated SNP rs12144978. This SNP has a strong CTCF peak nearby, is located in an intergenic region, and was identified to be involved in looping in five independent chromatin interaction datasets (Fig. 6a). Hi-C data identified two high confidence risk loops (220 kb and 320 kb) anchored by the PCa risk-associated CTCF site; each loop has convergent CTCF peaks at the anchor regions (Fig. 6b, c). Both loops were identified in prostate Hi-C datasets as well as in GM12878 Hi-C and HiChIP datasets and can be visually observed in the Hi-C interaction map (blue circles in Fig. 6b). Due to the higher resolution of the GM12878 Hi-C dataset, the genomic locations of the anchor regions of the two high confidence risk loops were taken from the GM12878 data. We note that there are additional CTCF sites near rs12144978. However, the other CTCF sites are 10 kb away from the anchor region and therefore were not identified as being involved in statistically significant loops with the prostate cancer risk-associated CTCF site; a browser snapshot of the CTCF ChIP-seq data and the loops identified by Hi-C can be seen in Fig. 10 and Additional file 1: Figure S3. Guide RNAs were introduced into 22Rv1 prostate cancer cells along with Cas9, and clonal populations were analyzed to identify clones in which both chr1 alleles were deleted for a 1607-bp region encompassing CTCF site 1. Using RNA-seq analysis of the clonal population, we found that deletion of the anchor region harboring CTCF site 1 caused a large increase (almost 100-fold) in expression of *KCNN3* (Fig. 6d), which is located within the loops anchored by the PCa risk-associated CTCF site. Other genes within the same loops or within ± 1 Mb from the risk CTCF site did not exhibit large changes in expression. However, other genes in the genome did show changes in expression, most likely as an indirect effect of altered expression of the nearby *KCNN3* gene (Additional file 1: Figure S2 and Additional file 6: Table S5). To determine if deletion of the region encompassing CTCF site 3, which anchors the larger loop but does not have a PCa risk-associated SNP nearby, also affected expression of *KCNN3*, we created clonal 22Rv1 cell populations having homozygous deletion of a 913-bp region encompassing CTCF site 3. RNA-seq analysis revealed a modest increase in the expression of *KCNN3* in cells homozygously deleted for site 3 (Fig. 6e). These data suggest that perhaps *KCNN3* expression is regulated by maintaining its topological associations within either the 220-kb or the 320-kb loop. If so, then deletion of the regions encompassing both sites 2 and 3 may be required to see the same effect on *KCNN3* expression as seen upon deletion of site 1. To test the effects of deletion of individual vs. multiple CTCF sites on *KCNN3* gene expression, we introduced guide RNAs (plus Cas9) to the regions encompassing CTCF sites 1, 2, or 3 individually, or guide RNAs targeting a combination of the regions, into 22Rv1 cells, harvested the transfected cell pools, and then performed RT-qPCR to measure *KCNN3* gene expression (Fig. 7). Introduction of the guide RNAs to delete a 1607-bp or 1221-bp region encompassing CTCF site 1 elicited a 90-fold increase in *KCNN3* expression, similar to the RNA-seq result shown in Fig. 6. Deletion of a 913-bp region encompassing site 3 showed a modest (less than 2-fold) increase in expression of KCNN3 (similar to the RNA-seq results); similar results were seen upon deletion of a 395-bp region encompassing site 2. Notably, the combination of site 2 and 3 deletions did not cause a large increase in *KCNN3* expression (~ 7-fold). Rather, only when the region encompassing CTCF site 1 (which we identified as a PCa risk-associated CTCF site) was deleted alone, or in combination with site 3, did *KCNN3* expression increase 100-fold.

**Fig. 8:**
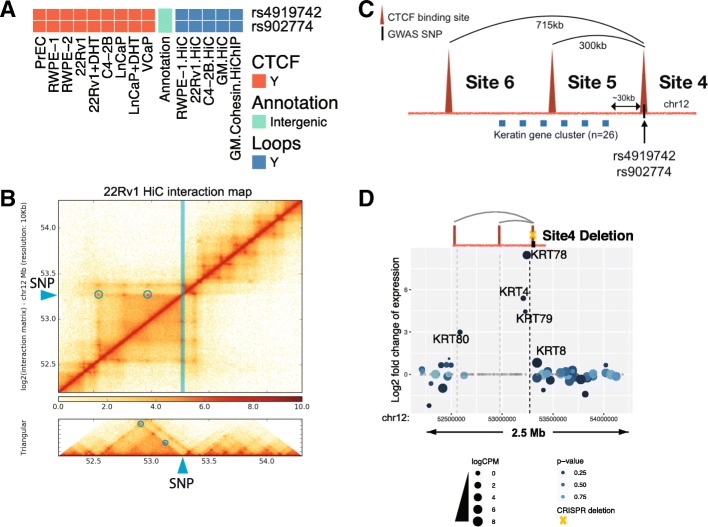
Deletion of the region encompassing the PCa risk-associated CTCF site near rs4919742 increases KRT gene expression. **a** A blowup of the CTCF peak information, genomic annotation, and looping information for rs4919742 from Fig. 4. **b** Hi-C chromatin interaction map of the region of chromosome 1 near the rs4919742. The location of the SNP is indicated by the blue line and arrow. The blue circles indicate the high confidence risk loops used in the analysis. **c** Detailed schematic of the high confidence risk loops in which rs4919742 is involved, as identified by the Hi-C chromatin interaction data; there are 26 keratin genes within the loops. **d** Shown is the fold-change expression of all genes within a ± 1-Mb region near rs4919742 in the cells deleted for a 2875-bp region encompassing the PCa risk-associated CTCF site (site 4); a volcano plot illustrating the genome-wide analysis of the RNA-seq data can be found in Additional file 1: Figure S2. The yellow X indicates which CTCF site has been deleted

**Fig. 9:**
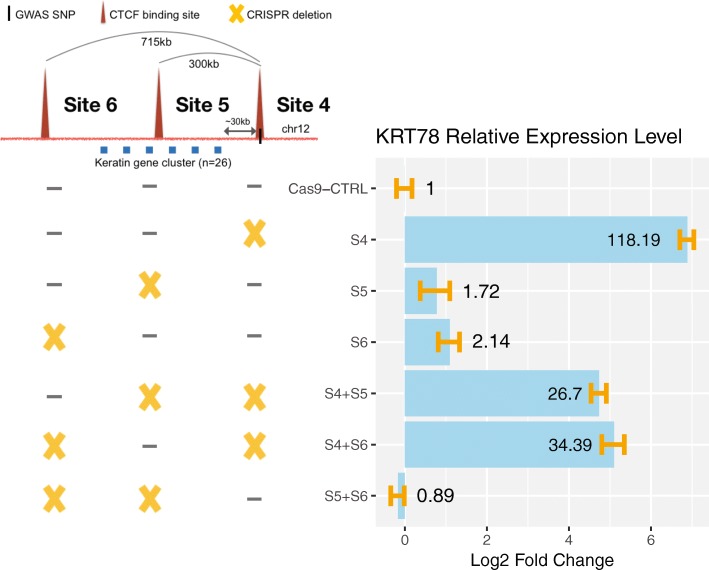
Analysis of the rs4919742-associated chromatin loops. Guide RNAs targeting regions encompassing CTCF site 4 (the PCa risk-associated CTCF site), CTCF site 5, and/or CTCF site 6 (or the empty guide RNA vector as a control) were introduced into 22Rv1 prostate cancer cells, along with Cas9. Cell pools were harvested, and *KRT78* expression was analyzed by RT-qPCR. Shown within the blue bars is the fold change in *KRT78* expression in the pools that received guide RNAs vs the vector control. The yellow X indicates which CTCF site has been deleted; the size of each deletion can be found in Additional file 5: Table S4

**Fig. 10:**
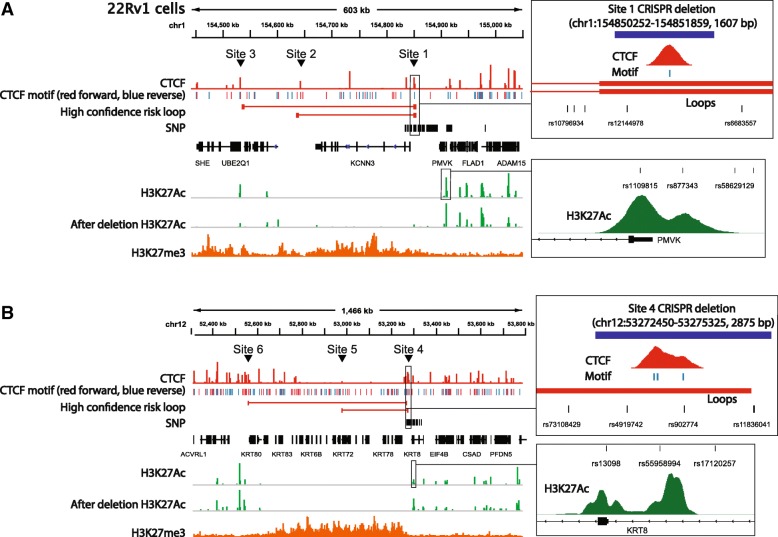
PCa risk-associated CTCF loops encompass enhancer deserts. Shown are genome browser snapshots of CTCF, CTCF motifs with orientation, H3K27Ac, and H3K27me3 ChIP-seq data for the regions near chromatin loops associated with the rs12144978 (**a**) or rs4919742 (**b**) risk SNPs. In each panel, the H3K27Ac ChIP-seq track for cells deleted for the region encompassing the PCa risk-associated SNP is also shown. Also shown are all fine-mapped SNPs in each locus and the high confidence risk loops identified by Hi-C chromatin interaction data anchored by each SNP and the RefSeq gene track. Insets show blowups of the regions containing the PCa risk-associated CTCF sites and PCa risk-associated H3K27Ac sites at each locus

We next assayed the effects of deletion of the region encompassing the CTCF site on chr12 near the PCa risk-associated SNP rs4919742. This PCa risk-associated CTCF peak is also located in an intergenic region and was identified to be involved in looping in five independent chromatin interaction datasets (Fig. 8a). Hi-C data identified two loops (300 kb and 715 kb) anchored by the PCa risk-associated CTCF site; each loop has convergent CTCF peaks at the anchors (Fig. 8b, c). Similar to the loops at CTCF site 1, both loops at CTCF site 4 were identified in prostate Hi-C datasets as well as in GM12878 Hi-C data and can be visually observed in the Hi-C interaction map (blue circles in Fig. 8b). Due to the higher resolution of the GM12878 Hi-C dataset, the genomic locations of the anchor regions of the two high confidence risk loops were taken from the GM12878 data. We note that there are additional CTCF sites near rs4919742. However, the other sites were not identified to be in statistically significant high confidence loops linked to the prostate cancer risk-associated CTCF site 4; a browser snapshot of the CTCF ChIP-seq data and the loops identified by Hi-C can be seen in Fig. 10 and Additional file 1: Figure S4. Guide RNAs were introduced into 22Rv1 prostate cancer cells along with Cas9, and clonal populations were analyzed to identify clones in which both chr12 alleles were deleted for a 2875-bp region encompassing CTCF site 4. We found that deletion of this region caused a large increase in expression of *KRT78*, *KRT4*, *KRT79*, and *KRT80* (Fig. 8d). *KRT78*, *KRT4*, and *KRT79* are located within the 300-kb loop whereas *KRT80* is outside of the 300-kb loop but within the larger 715-kb loop, both of which are anchored by the PCa risk-associated CTCF site 4. To test the effects of deletion of individual vs. multiple CTCF sites on KRT gene expression, we introduced guide RNAs (plus Cas9) to regions encompassing CTCF sites 4, 5, or 6 individually, or guide RNAs that target a combination of the sites, into 22Rv1 cells, harvested the transfected cell pools, and then performed RT-qPCR to measure *KRT78* gene expression (Fig. 9). Introduction of guide RNAs that would delete a 2875-bp or a 1384-bp region encompassing PCa risk-associated CTCF site 4 showed more than a 100-fold increase in *KRT78* expression, similar to the RNA-seq analyses shown in Fig. 8. Deletion of 1969-bp and 5457-bp regions encompassing CTCF sites 5 or 6, respectively (which are not associated with PCa), showed very modest increases in expression of *KRT78*, whereas the combination of deletion of sites 5 and 6 did not increase *KRT78* expression. The only large changes in *KRT78* expression were in cells deleted for the region encompassing CTCF site 4 alone or when deleted in combination with other CTCF sites.

Finally, we investigated the cell-type specificity of the response to deletion of the regions encompassing the PCa risk-associated CTCF sites by also deleting these regions in HEK293T kidney cells and HAP1 chronic myelogenous leukemia cells. Although the *KRT78* gene was upregulated (~ 25-fold) in both HEK293T and HAP1 when a 1.6-kb region encompassing PCa risk-associated CTCF site on chr12 was deleted (Additional file 1: Figure S3), deletion of a 2.8-kb region encompassing the PCa risk-associated CTCF site on chr1 in HEK293T or HAP1 cells did not result in an increase in *KCNN3* expression (Additional file 1: Figure S4).

### PCa risk-associated CTCF loops may sequester genes from enhancers located outside the loops

To gain insight into the mechanism by which the PCa risk-associated CTCF sites near SNPs rs12144978 and rs4919742 may regulate expression of *KCNN3* and *KRT78*, respectively, we examined the pattern of H3K27Ac peaks in a large region surrounding each SNP (Fig. 10). Interestingly, in both cases, the genomic regions within the loops that are anchored by the PCa risk-associated SNP are devoid of the active enhancer mark H3K27Ac. These are very large genomic regions (~ 200–600 kb) to lack any H3K27Ac peaks. This pattern suggested two mechanisms by which these CTCF sites could potentially maintain expression of *KCNN3* and *KRT78* at low levels. First, the loops may prevent activation of potential enhancers by formation of a repressive chromatin structure. We determined that the loop regions anchored by the two PCa risk-associated CTCF sites (site 1 on chr1 and site 4 on chr12) are both covered by H3K27me3, which is known to be associated with polycomb-mediated gene silencing [33]; deletion of the risk-associated CTCF sites may result in the formation of new enhancers within these previously repressed regions. Alternatively, the PCa risk-associated CTCF sites may prevent the promoters of the *KCNN3* and *KRT78* genes from interacting with a pre-existing active enhancer(s) located outside the loop (in this case, the enhancer would be marked by H3K27Ac in both control and CRISPR-deleted cells). To distinguish these possibilities, we performed H3K27Ac ChIP-seq in clonal population of cells homozygously deleted for either the PCa risk-associated CTCF site 1 on chr1 or the site 4 on chr12. Interestingly, we found that the regions remained as enhancer deserts, even after deletion of the PCa risk-associated CTCF sites. Our data supports a model in which the PCa risk-associated CTCF-mediated loops insulate the *KCNN3* and *KRT78* promoters from nearby pre-existing active enhancers.

## Discussion

We performed a comprehensive analysis of the regulatory potential of 2,181 fine-mapped PCa risk-associated SNPs, identifying a subset of these SNPs that fall within DHS sites located within either a H3K27Ac peak or a CTCF peak defined by ChIP-seq datasets we produced for normal and tumor prostate cells. After selecting the fine-mapped SNPs that fall within these active regulatory regions, we next identified the subset of SNPs that lie within an anchor region of a chromatin loop, using in situ Hi-C data from normal and tumor prostate cells. Using this information, we predicted a set of target genes that are regulated by PCa risk-related H3K27Ac-marked enhancers (Additional file 7: Table S6). Finally, we used CRISPR-mediated deletion to remove CTCF anchor regions that encompass PCa risk-associated CTCF sites and also deleted regions encompassing CTCF sites that fall within the anchor regions of the other ends of the loops. We found that deletion of the region encompassing a PCa risk-associated CTCF site on chr1 or the region encompassing a PCa risk-associated site on chr12 turns on a nearby gene located in an enhancer desert. Our results suggest that these two PCa risk-associated CTCF sites may function by encaging cancer-relevant genes in repressive loops.

We focused our studies on two PCa risk-associated genomic loci (one on chr1 and one on chr12), each of which harbors a CTCF site which is both near a SNP identified by fine-mapping to be related to increased risk for PCa and identified by in situ Hi-C analysis to be involved in large chromatin loops. Upon deletion of the region encompassing the PCa risk-associated CTCF site on chr1, we found that *KCNN3* expression was increased ~ 100-fold; no other genes within ± 1 Mb of the risk CTCF site on chr1 showed a large change in gene expression. Similarly, deletion of the region encompassing the risk-associated CTCF site on chr12 caused a ~ 100-fold increase in expression of *KRT78*; in this case, four of the other nearby KRT genes also showed increased expression, albeit not as high as *KRT78*. The very large increases in gene expression that we observed upon deletion of regions encompassing PCa risk-associated CTCF sites are interesting due to the fact that removal of CTCF or the cohesin component RAD21 from the cell has quite modest overall effects on the transcriptome. Nora et al. [34] identified only a small number of genes (~ 200) that were upregulated more than 10-fold after removal of CTCF from mES cells using an auxin degron system. The authors noted that not all genes within a TAD responded in the same way to CTCF depletion and concluded that depletion of CTCF triggers upregulation of genes that are normally insulated from neighboring enhancers by a TAD boundary. Similarly, Rao et al. [35] found that auxin-mediated depletion of RAD21 (a core component of cohesin) in HCT116 colon cancer cells led to the upregulation of a small number of genes (~ 200 genes showed at least a 30% increase in expression). These analyses of the transcriptional consequences of CTCF or RAD21 depletion are similar to our studies of CRISPR-mediated CTCF site deletion. However, the degree of upregulation that we observed upon deletion of the regions encompassing PCa risk-associated CTCF sites is much greater than the majority of the effects observed in the previous studies.

As noted above, we observed profound effects on gene expression when we deleted regions encompassing CTCF sites related to increased risk for PCa. To investigate whether other nearby CTCF sites are also involved in regulating gene expression, we also deleted two additional CTCF sites on chr1 and two additional CTCF sites on chr12 that are at the other end of the chromatin loops formed by the risk-associated CTCF sites. We found that on both chr1 and chr12, deletion of either one of the CTCF sites that pair with the PCa risk-associated CTCF site had little effect on gene expression. One might expect that simultaneous deletion of both of the pairing CTCF anchors would cause an increase in gene expression. However, single deletion of the region encompassing the PCa risk-associated CTCF site had much greater effects on expression than simultaneous removal of the other two sites. These results demonstrate that the increased expression of *KCNN3* and *KRT78* is not simply a response to the method of CRISPR-mediated deletion but rather suggest that the regions encompassing the PCa risk-associated CTCF sites are more important in regulating the expression of these genes than are the CTCF sites at the other end of the loops. Perhaps the PCa risk-associated CTCF sites can establish repressive loops with other CTCF sites upon deletion of the other ends of the original loops; we note that there are several CTCF peaks with motifs oriented in the correct direction that could possibly be adopted as a new anchor for CTCF site 1 and site 4 if the normal loop anchor sites are deleted. Also, it is possible that other, lower frequency interactions encompassing *KCNN3* or *KRT78* (involving CTCF site 1 or site 4, respectively) also create repressive loops (see Additional file 8: Table S7 for a list of all loops involving CTCF site 1 and site 4). Finally, it is also possible that other transcription factors that bind to sequences near CTCF site 1 or site 4 (within the regions targeted for deletion) serve as repressors of the *KCNN3* and *KRT78* promoters. In this case, CTCF-mediated looping may not be the primary mechanism by which the expression of two genes is kept at low levels.

**Fig. 11:**
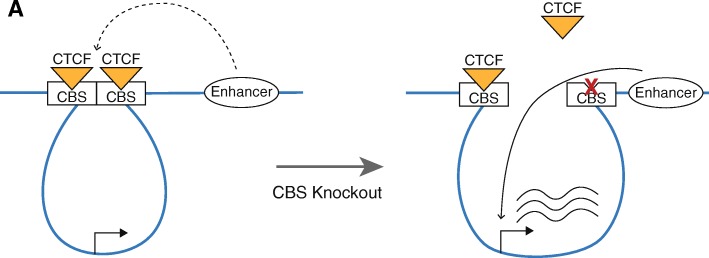
PCa risk-associated CTCF loops may sequester genes from enhancers located outside the loops. Shown is one potential model for gene activation that occurs upon deletion of a PCa risk-associated CTCF site. In this model, the entire CTCF-binding site (CBS) is removed and therefore the loop is broken, allowing an enhancer outside the original loop to increase the activity of a promoter located within the original loop

Both *KCNN3* and *KRT78* are each located within large genomic regions that are devoid of the H3K27Ac mark. The upregulation of *KCNN3* and *KRT78* upon deletion of the risk-associated CTCF regions could be due to the creation of new active enhancers in the previous enhancer deserts, which are covered by the repressive H3K27me3 mark in control cells. Alternatively, it has previously been proposed that CTCF can limit gene expression by sequestering a gene within a loop and preventing it from being regulated by nearby enhancers [36, 37]. Therefore, it was possible that pre-existing enhancers, located outside the enhancer deserts, gain access to the promoters of *KCNN3* and *KRT78* genes after deletion of the regions encompassing the risk CTCF sites (i.e., an enhancer adoption model). H3K27Ac ChIP-seq analysis of clonal cell populations homozygously deleted for the regions encompassing the risk-associated CTCF sites showed that new active enhancers are not created within the large enhancer deserts (Fig. 10). Therefore, it is likely that the increased expression of *KCNN3* and *KRT78* is due to adoption of an existing enhancer, not creation of a new enhancer (Fig. 11). We note that not all nearby genes are upregulated when the regions encompassing the PCa risk-associated CTCF sites are deleted. This suggests that there may be some biochemical compatibility between enhancers and promoters that is required for robust activation and/or that other factors that prime a specific promoter for activation must be present. Interestingly, through our analysis of H3K27Ac sites associated with PCa risk (Fig. 3), we have identified an H3K27Ac site overlapping several PCa risk-associated SNPs that is ~ 70 kb from the *KCNN3* transcription start site (Fig. 10a) and an H3K27Ac site overlapping several PCa risk-associated SNPs that is ~ 60 kb upstream from the *KRT78* transcription start site (Fig. 10b). We note that in each case, the PCa risk-associated H3K27Ac site is the closest H3K27Ac site to the deleted CTCF site and is the first H3K27Ac at the edge of the enhancer desert. Thus, these PCa risk-associated H3K27Ac sites may be involved in “enhancer adoption” by the promoters of the *KCNN3* and *KRT78* genes in the cells deleted for the PCa risk-associated CTCF sites.

Although the effects of deletion of other GWAS-related CTCF sites have not been reported, Gallager et al. have proposed that a CTCF site near a SNP involved in risk for frontotemporal lobar degeneration creates a loop that enhances expression of *TMEM106B*; however, because the CTCF site was not deleted, the actual effect of the site on gene expression is not known [38]. Several groups have studied other disease-related CTCF sites [39]. In most cases, the CTCF sites have resided within a TAD boundary element and, when these sites are deleted, modest upregulation of a nearby gene has occurred. For example, deletion of a TAD boundary was shown to increase expression of *PAX3*, *WNT6*, and *IHH* [40] via a proposed mechanism of enhancer adoption made possible by removal of a repressive loop. Enhancer adoption has also been linked to AML/MDS, MonoMAc/Emerger syndromes, and medulloblastoma [41, 42]. Also, investigators have shown that elimination of a boundary site of an insulated neighborhood can modestly activate expression of an oncogene [43, 44]. Other examples of enhancer adoption include a modest upregulation of the *Fnb2* gene when a CTCF site located 230 kb downstream is deleted [30] and a 3-fold increase in *PDGFRA* expression upon deletion of a CTCF site [37]. Interestingly, Ibn-Salem et al. searched the Human Phenotype Ontology database and identified 922 deletion cases in which tissue-specific enhancers have been brought into the vicinity of developmental genes as consequence of a deletion that removed a TAD boundary. They predicted that 11% of the phenotype effects of the deletions could be best explained by enhancer adoption that occurs upon removal of TAD boundary [45]. Future studies that test these predictions would help to understand the global significance of repressive 3D chromatin loops.

## Conclusions

We have identified PCa risk-associated CTCF anchor regions that appear to function by creating a repressive regulatory environment; deletion of these anchor regions results in a very large increase (~ 100-fold) in expression of *KCNN3* (upon deletion of the CTCF site on chr1) or *KRT78* (upon deletion of the CTCF site on chr12). A link between KCNN3, also known as SK3, and prostate cancer biology has been previously observed. KCNN3 is a calcium-activated potassium channel that has been shown to enhance tumor cell invasion in breast cancer and malignant melanoma [46]. For example, Chantome et al. [47] have shown that the majority of breast and prostate cancer samples from primary tumors or bone metastases (but not normal tissues) are positive for KCNN3. Of note, the shRNA-mediated reduction of KCNN3 RNA did not result in changes in cell proliferation, but rather resulted in a lower number of bone metastases in a nude mouse model system. The bone is the most frequent site of prostate carcinoma metastasis with skeletal metastases identified at autopsy in up to 90% of patients dying from prostate carcinoma [48–50]. Taken together with previous studies, our work suggests that binding of CTCF to rs12144978 may, via its repressive role on KCCN3 expression, play a protective role regarding human prostate cancer. Of clinical relevance, edelfosine, a glycerophospholipid with antitumoral properties that inhibits SK3 channel activity, can inhibit migration and invasion of cancer cells in vitro and in vivo in an SK3-dependent manner, pointing towards a possible use of edelfosine in prostate cancer treatment [51–54]. Although KRT78 has not previously been associated with prostate cancer, it has been identified as a diagnostic marker for metastatic melanoma [55] and cervical cancers [56]. Investigation of the function of other GWAS-identified CTCF sites involved in chromatin loops may reveal additional genes involved in the development or diagnosis of prostate cancer.

## Methods

### Cell culture

C4-2B cells were obtained from ViroMed Laboratories (Minneapolis, MN, USA). RWPE-1 (CRL-11609), RWPE-2 (CRL-11610), 22Rv1 (CRL-2505), LNCaP (CRL-1740), and VCap (CRL-2876) were all obtained from American Type Culture Collection (ATCC). The human normal prostate epithelial cells (PrEC) were obtained from Lonza (CC-2555, Lonza, Walkersville, MD, USA). Cells were cultured according to the suggested protocols at 37 °C with 5% CO_2_. The medium used to culture C4-2B (RPMI 1640), VCaP (DMEM), LNCaP (RPMI 1640), and 22Rv1 (RPMI 1640) was supplemented with 10% fetal bovine serum (Gibco by Thermo Fisher, #10437036) plus 1% penicillin and 1% streptomycin. For DHT experiments, 22Rv1 and LNCaP cells were grown in phenol-red free RPMI 1640 with 10% charcoal-stripped fetal bovine serum for 48 h and then treated with 10 nM DHT or vehicle for 4 h before harvest. RWPE-1 and RWPE-2 cells were cultured in Keratinocyte Serum Free Medium kit (Thermo Fisher Scientific, 17005-042) without antibiotics. PrEC cells were grown using the PrEGM Bullet Kit (Lonza, #CC-3166). All cell lines were authenticated at the USC Norris Cancer Center cell culture facility by comparison to the ATCC and/or published genomic criteria for that specific cell line; all cells were documented to be free of mycoplasma. Pre-authentication was performed at Lonza (Walkersville, MD, USA) for PrEC. Detailed cell culture protocols are provided for each cell line/primary cells in Additional file 9: Cell Culture Protocols.

### ChIP-seq

All ChIP-seq samples were performed in duplicate according to a previously published protocol [57–59]. Five micrograms of CTCF antibody (Active Motif #61311) was used to precipitate 20 μg chromatin for 22Rv1, PrEC, RWPE-2, VCaP (rep1) cells, and 10 ul CTCF antibody (Cell Signaling #3418S) were used to precipitate 20 μg chromatin for LNCaP, C4-2B, RWPE-1, VCaP (rep2) cells. Eight micrograms of H3K27Ac antibody (Active Motif #39133) was used to precipitate 20 μg chromatin for all H3K27Ac ChIP-seq. Ten microliters of H3K27me3 antibody (Cell Signaling #9733S) was used to precipitate 20 μg of 22Rv1 chromatin for K27me3 ChIP-Seq. All antibodies were validated according to ENCODE standards; validation documents are available on the ENCODE portal (encodeproject.org). ChIP-seq libraries were prepared using Kapa Hyper prep kit (Kapa #KK8503) according to the provided protocol. Samples were sequenced on Illumina HiSeq3000 machine using paired-ended 100-bp reads (except for H3K27Ac-LNCaP ChIP-seqs which were sequenced using 50-bp single-ended reads). All ChIP-seq data were mapped to hg19, and peaks were called using MACS2 [60] after preprocessing data with the ENCODE3 ChIP-seq pipeline (https://www.encodeproject.org/chip-seq/). High confidence (HC) peaks (Additional file 3: Table S2) were called by taking peaks that were found in both duplicates for a given cell line/antibody combination using intersectBed function from the bedtools suite [61].

### Hi-C

In situ Hi-C experiments were performed following the original protocol by Rao et al. [25] with minor modifications [26]. Hi-C datasets were processed using the HiC-Pro [62] to make normalized 10-kb resolution matrices. Intra-chromosomal loops (50 kb to 10 Mb range) were selected using Fit-Hi-C using a *q* value < 0.05 [63], as we have described in previous studies [26]. Hi-C chromatin interaction heatmaps were visualized using the HiCPlotter [64].

### SNP annotation

Fine-mapped SNPs from previous studies [8–10] were curated, and SNP information was extracted from dbSNP147. SNPs were annotated (Additional file 4: Table S3) by their overlap with the genomic coordinates of (a) a comprehensive set of DHS downloaded from the ENCODE project portal at encodeproject.org, (b) H3K27Ac high confidence peaks, (c) regions corresponding to ± 1 kb from CTCF high confidence peak summits, and (d) chromatin loops and topologically associated domains from Hi-C or Cohesin HiChIP data from GM12878 cells [25, 27], RWPE-1 normal prostate cells [26], and 22Rv1 and C4-2B prostate cancer cells (Rhie et al., in preparation); annotation was performed using the annotateBed function in bedtools [61].

### CRISPR/Cas9-mediated genomic deletions

gRNAs were cloned into pSpCas9(BB)-2A-Puro (PX459) V2.0 plasmid (Addgene #62988) following the previously published protocol [65]; the sequence of all guide RNAs used in this study can be found in Additional file 5: Table S4. 22Rv1 cells (wild type or single deletion clones) were transfected with guide RNA and Cas9 expression plasmids using Lipofectamine LTX with PLUS regent (Thermo Fisher, #15338100) according to the manufacture’s protocol. After 24 h transfection, cells were treated with 2 μg/mL puromycin for 48–72 h (ensuring that the un-transfected control cells all died). The media was then replaced with new media without puromycin, and the cells were allowed to recover for 24–48 h. The cells were then harvested for further analysis or disassociated and sorted into 96-well plates with 1 cell/well using flow cytometry. The single cells were grown into colonies, then expanded to obtain clonal populations for further analysis. Cell pools and single cells were harvested using QuickExtract DNA Extraction Solution (Epicentre #QE9050) according to the manufacture’s protocol and genotyped by PCR using primers listed in Additional file 5: Table S4.

### RNA analyses

Total RNA was extracted from cell pools and cell populations derived from single cell colonies using TRIzol protocol (Thermo Fisher, #15596026) or DirectZol (Zymo, #R2062). For RNA-seq, ERCC spike-in control mix 1 (Thermo Fisher, #4456704) was added before library preparation, according to the manufacturer’s suggestion. Libraries were made using the Kapa Stranded mRNA kit with beads (Kapa #KK8421). Samples were sequenced on an Illumina HiSeq3000 with single-end 50-bp read length. RNA-seq results were aligned to Gencode v19, and reads were counted using STAR [66]. Differentially expressed genes were determined using edgeR [67, 68], and batch effects were corrected using the RUVg function of RUVseq [69]. See Additional file 2: Table S1 for more information about the RNA-seq libraries and Additional file 6: Table S5 for the list of genes differentially expressed in cells harboring deletions of PCa risk-associated CTCF sites. For analysis of RNA from cell pools, cDNA libraries were made using the Maxima kit (Thermo Fisher, #K1671). qPCR was performed using SYBR Green (Bio-Rad, #1725275) and a Bio-Rad CFX96 machine (Bio-Rad, #1855196). See Additional file 5: Table S4 for information concerning the primers used in RT-qPCR reactions.

For the analysis of site 1 by RNA-seq, a 1607-bp region was deleted using guide RNAs 11+12; two independent clones were identified, and each clone was analyzed in triplicate (Fig. 6). The effects of deleting site 1 on the expression of *KCNN3* were also analyzed in a cell pool using guide RNAs 11+12 or 35+36 (which deleted a 1221-bp region encompassing site 1), in wt cells and in a cell pool that was previously deleted for a 913-bp region encompassing site 3 (Fig. 7). The effects of deleting site 2 on the expression of *KCNN3* were analyzed in a cell pool using guide RNAs 24+26 (which deleted a 395-bp region encompassing site 2), in wt cells and in a cell clone previously deleted for site 3 (Fig. 7). For the analysis of site 3 deletion by RNA-seq, a 913-bp region was deleted using guide RNAs 5+6; three independent clones were identified, and each clone was analyzed by RNA-seq. The effects of deleting site 3 in combination with deletions of site 1 and site 2 are described above. For the analysis of site 4 by RNA-seq, a 2875-bp region was deleted using guide RNAs 22+23; two independent clones were identified and each clone was analyzed in triplicate by RNA-seq (Fig. 8). The effects of deleting site 4 on the expression of *KRT78* were also analyzed in a cell pool using guide RNAs 21+37 to delete a 1384-bp region encompassing site 4 plus guide RNAs 40+41 to delete a 1969-bp region encompassing site 5 or guide RNAs 38+39 to delete a 5457-bp region encompassing site 6 (Fig. 9). The effects of deleting site 5 on *KRT78* expression was analyzed using guide RNAs 40+41 alone or in combination with guide RNAs 38+39 to delete site 6. Finally, the effects of deleting a 5457-bp region encompassing site 6 on *KRT78* expression was analyzed in a cell pool using guide RNAs 38+39 (Fig. 9); combination deletions are described above; see Additional file 5: Table S4 for details of all guide RNA locations and deletion sizes.

## Additional files

Additional file 1: **Figure S1.** High confidence ChIP-seq peaks. Figure S2. Genome-wide RNA-seq analysis of cells deleted for PCa risk-associated CTCF sites. Figure S3. Deletion of PCa risk-associated CTCF site 1 in different cell lines. Figure S4. Deletion of PCa risk-associated CTCF site 4 in different cell lines. (PDF 2538 kb)

Additional file 2: **Table S1.** List of ChIP-seq and RNA-seq datasets. 18 KB (XLSX 17 kb)

Additional file 3: **Table S2.** ChIP-seq peaks. (XLSB 21665 kb)

Additional file 4: **Table S3.** Annotated SNPs. (XLSX 12 kb)

Additional file 5: **Table S4.** Sequences of guide RNAs and primers. (XLSX 15 kb)

Additional file 6: **Table S5.** RNA-seq analyses. (XLSX 4442 kb)

Additional file 7: **Table S6.** Predicted genes regulated by PCa-related K27Ac sites. (XLSX 11 kb)

Additional file 8: **Table S7.** 22Rv1 Hi-C interactions involving CTCF site 1 and CTCF site 4. (XLSX 12 kb)

Additional file 9: Cell culture protocols. (PDF 388 kb)

## Abbreviations

DHS: DNase hypersensitive site
DHT: Dihydrotestosterone
GWAS: Genome-wide association studies
PCa: Prostate cancer
SNP: Single nucleotide polymorphisms
TAD: Topological associating chromatin domain
