# Supplementary material for: CRISPR-mediated deletion of prostate cancer risk-associated CTCF loop anchors identifies repressive chromatin loops
Source: Genome Biol. 2018 Oct 8;19:160. doi: 10.1186/s13059-018-1531-0 (PMC6176514; doi:10.1186/s13059-018-1531-0)
Supplement: Supplementary file 1 — Figure S1. High confidence ChIP-seq peaks. Figure S2. Genome-wide RNA-seq analysis of cells deleted for PCa risk-associated CTCF sites. Figure S3. Deletion of PCa risk-associated CTCF site 1 in different cell lines. Figure S4. Deletion of PCa risk-associated CTCF site 4 in different cell lines. (PDF 2538 kb) [file 13059_2018_1531_MOESM1_ESM.pdf]

A

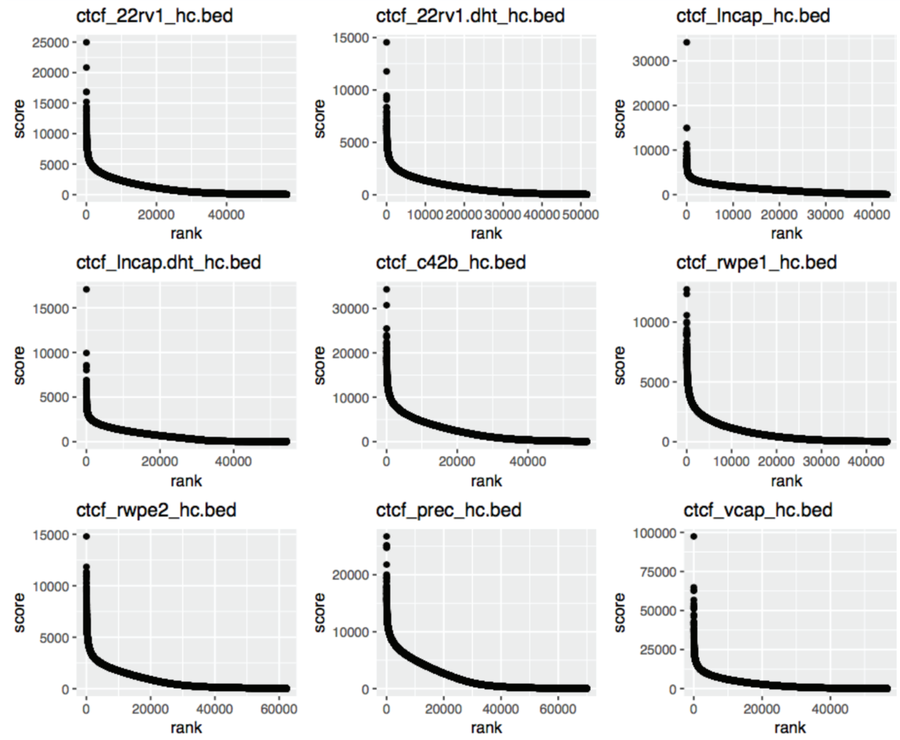

B

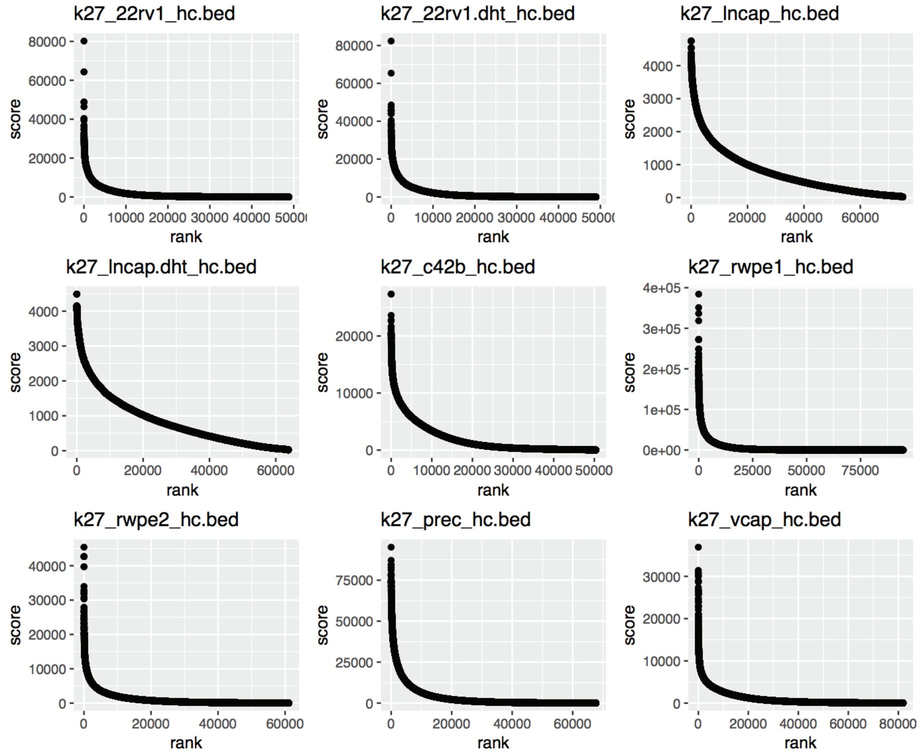

Figure S1. High confidence ChIP-seq peaks.
Shown are peak score vs. peak rank graphs for **(A)** H3K27Ac or **(B)** CTCF ChIP-seq datasets for normal (PrEC and RWPE1) and tumor (RWPE2, LnCaP, VCaP, 22RV1, and C4-2B) prostate cells; for LnCaP and 22RV1 cells, ChIP-seq was performed before and after addition of DHT.

Guo_Figure S1.

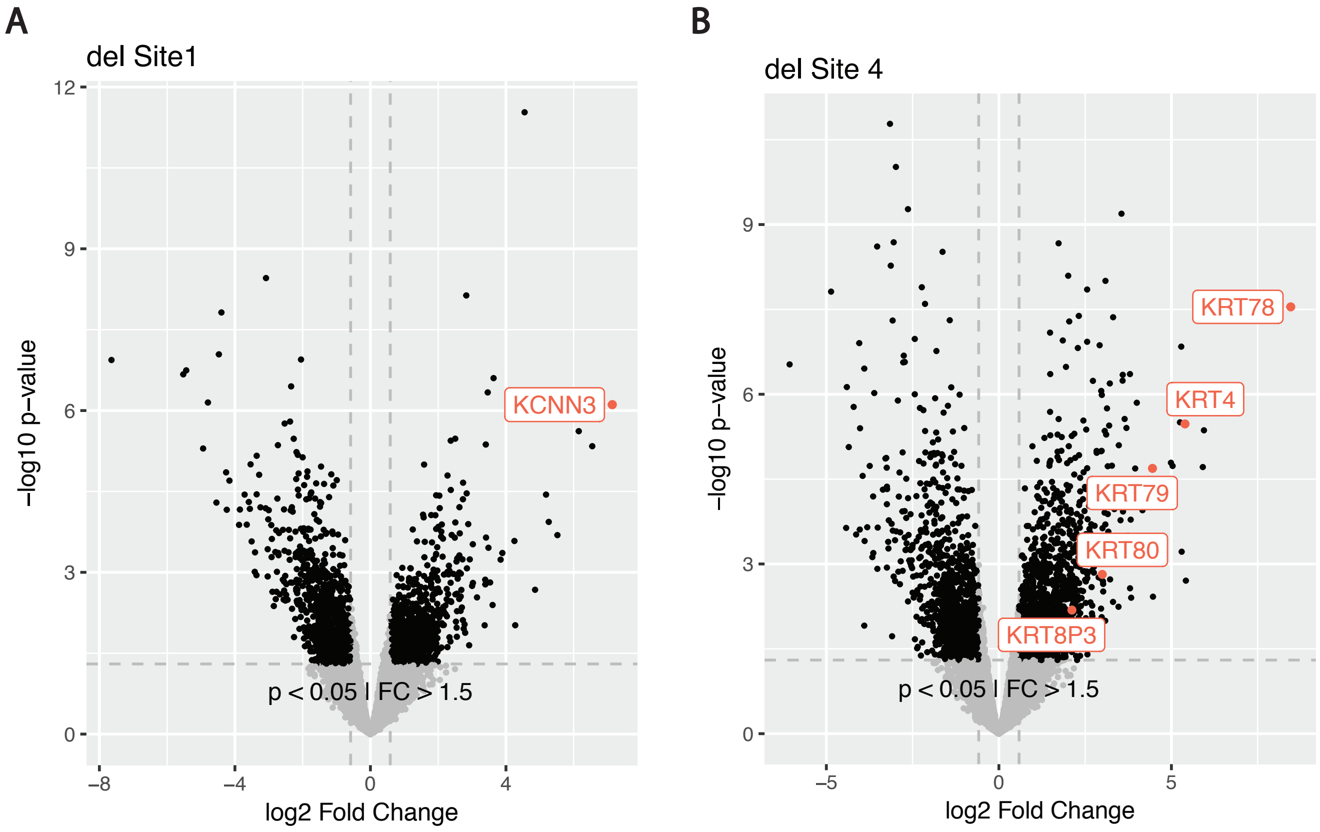

Figure S2. Genome-wide RNA-seq analysis of cells deleted for PCa risk-associated CTCF sites. Shown are volcano plots representing changes in gene expression, as compared to control 22Rv1 cells, in cells having a homozygous deletion of CTCF PCa risk-associated site 1 **(A)** or site 4 **(B)**.

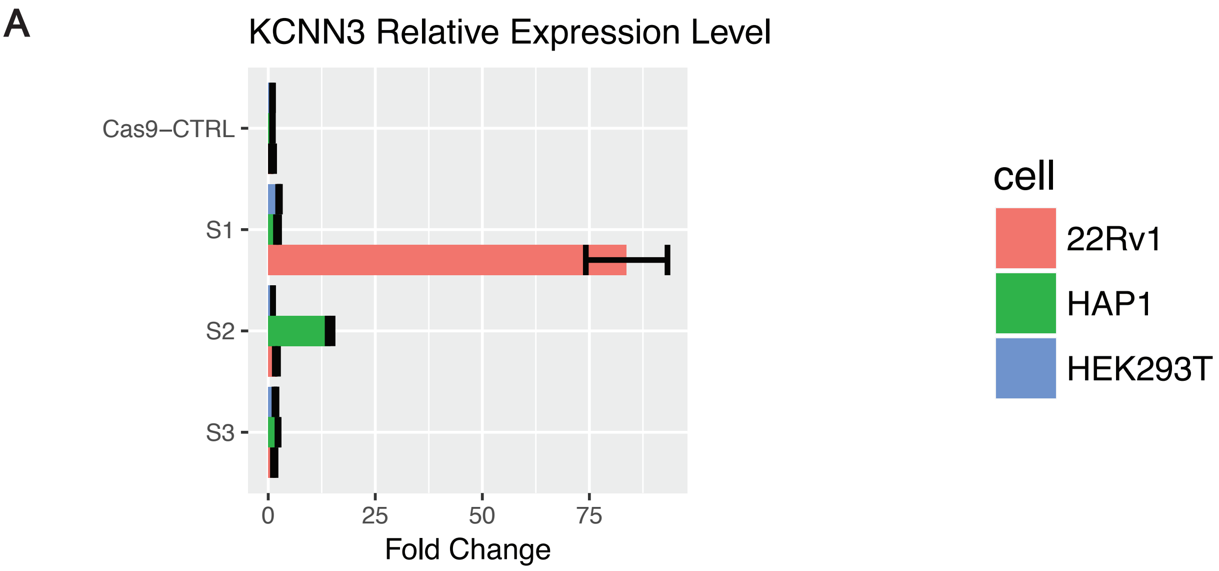

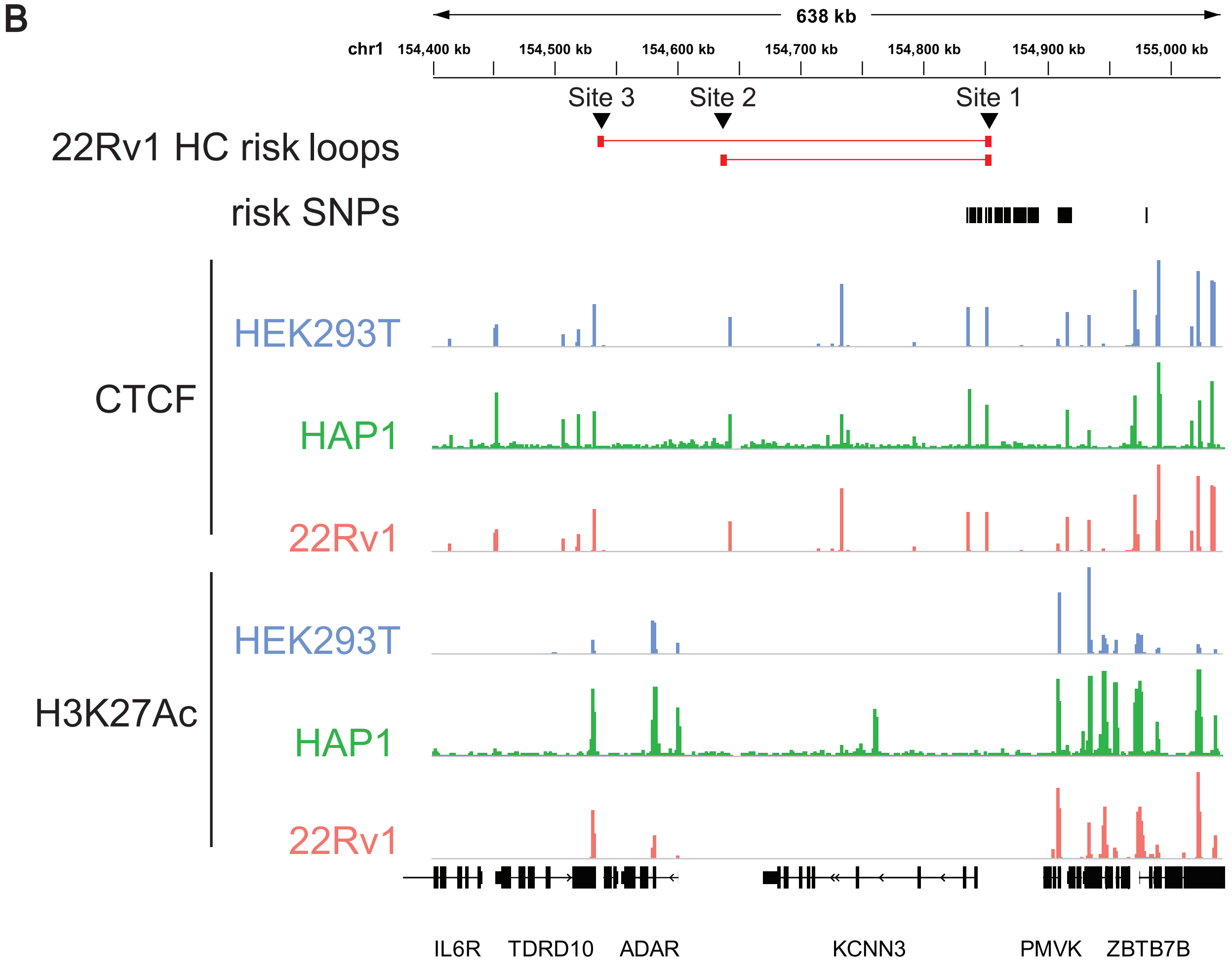

Figure S3. Deletion of PCa risk-associated CTCF site 1 in different cell lines.
(A) Shown is the expression level of KCNN3 in control and cell pools transfected with guide RNAs targeting CTCF site 1, for 22RV1, HAP1, and HEK293T cells. **(B)** Shown are CTCF and H3K27Ac ChIP-seq tracks for 22RV1, HAP1, and HEK293T cells.

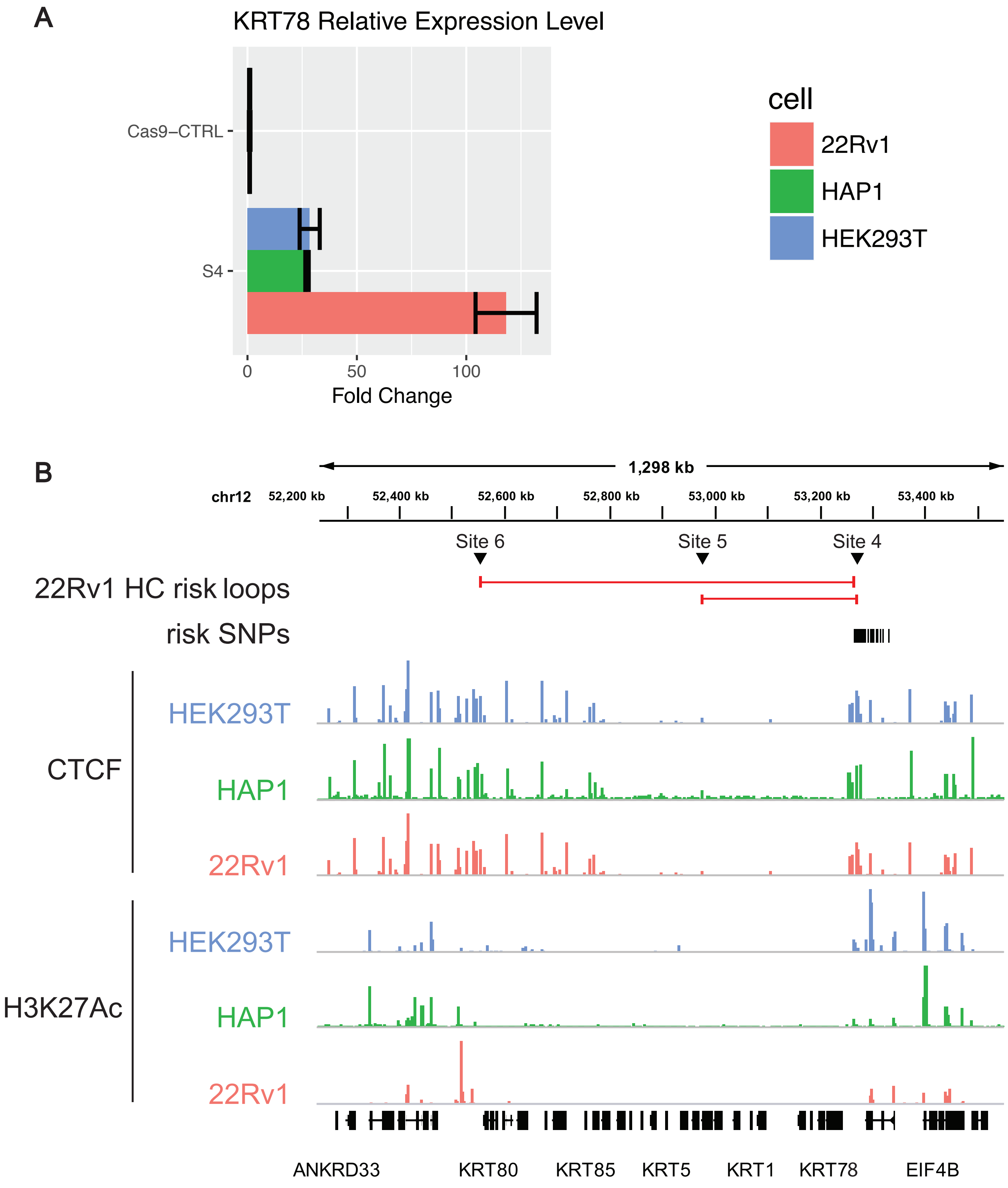

Figure S4. Deletion of PCa risk-associated CTCF site 4 in different cell lines.
(A) Shown is the expression level of KRT78 in control and cell pools transfected with guide RNAs targeting CTCF site 4, for 22RV1, HAP1, and HEK293T cells. **(B)** Shown are CTCF and H3K27Ac ChIP-seq tracks for 22RV1, HAP1, and HEK293T cells.
