# Supplementary material for: CRISPR-mediated deletion of prostate cancer risk-associated CTCF loop anchors identifies repressive chromatin loops
Source: Genome Biol. 2018 Oct 8;19:160. doi: 10.1186/s13059-018-1531-0 (PMC6176514; doi:10.1186/s13059-018-1531-0)
Supplement: Supplementary file 9 — Cell culture protocols. (PDF 388 kb) [file 13059_2018_1531_MOESM9_ESM.pdf]

C4-2B

Farnham Lab (150720 version)

1. Thaw the vial containing 1mL of C4-2B cells in a 37^oC waterbath. Transfer cells to a 10cm dish containing 9mL of growth medium (RPMI 1640 with 10% FBS and optional addition of penicillin (100 units/mL), streptomycin (100 μg/mL)).
2. Incubate in 5% CO_2 incubator at 37^oC overnight.
3. Discard medium carefully, add 10mL of fresh growth medium.
4. Grow cells until about 80% (+/-10%) confluence.
5. Wash cells carefully with warm PBS.
6. Trypsinize cells with 3mL of 0.05% trypsin and 0.02% EDTA at 37^oC for 5 min.
7. Neutralize trypsin with 7mL of fresh growth medium.
8. Spin down cells at 470rcf for 5 min at room temperature.
9. Discard supernatant, resuspend cells with 5mL of growth medium, transfer 5mL of cells to a 15cm dish containing 15mL of growth medium growth as new passage.
10. When cells grow to about 80% (+/-10%) confluence, harvest cells by using above steps 5 to 8.
11. Freeze cell stocks by using above steps 5 to 6 with 2x volumes when cells are in a 15cm dish. Discard supernatant, resuspend cells with 3mL of freeze medium (RPMI 1640 with 20% FBS and 10% DMSO), separate into 3 cryo vials, put in foam storage box in -80^oC overnight, then remove to liquid nitrogen.

VCaP (ATCC, Cat# CRL-2876)

Farnham Lab (150723 version)

Regular culture:

1. Thaw the vial containing 1mL of VCaP cells in a 37°C waterbath. Transfer cells to a 10cm dish containing 9mL of growth medium (DMEM with 10% FBS and optional addition of penicillin (100 units/mL), streptomycin (100 µg/mL)).
2. Incubate in 5% CO_2 incubator at 37°C overnight.
3. Discard medium carefully, add 10mL of fresh growth medium.
4. Grow cells until about 80% (+/-10%) confluence.
5. Wash cells carefully with warm PBS.
6. Trypsinize cells with 3mL of 0.05% trypsin and 0.02% EDTA at 37°C for 5 min.
7. Neutralize trypsin with 7mL of fresh growth medium.
8. Spin down cells at 470rcf for 5 min at room temperature.
9. Discard supernatant, resuspend cells with 5mL of growth medium, transfer 5mL of cells to a 15cm dish containing 15mL of growth medium growth as new passage.
10. When cells grow to about 80% (+/-10%) confluence, harvest cells by using above steps 5 to 8.
11. Freeze cell stocks by using above steps 5 to 6 with 2x volumes when cells are in a 15cm dish. Discard supernatant, resuspend cells with 3mL of freeze medium (DMEM with 20% FBS and 10% DMSO), separate into 3 cryo vials, put in foam storage box in -80°C overnight, then remove to liquid nitrogen.

LNCaP cell line ATCC # CRL-1740

The LNCaP cell line is an epithelial cell line derived from a human prostate carcinoma. LNCaP cells grow as a loosely adherent layer of cells that are prone to growth in clusters. Do not plate LNCaP cells too sparsely or they will not grow well. Under the conditions described below they double every 48-60 hours.

Cell culture

Cells are grown in RPMI 1640 media containing penicillin (100 units/ml), streptomycin (100 µg/ml), and 10% FBS at 37°C in 5% CO_2 incubator.

1. Thaw the vial containing LNCaP cells (ATCC # CRL-1740) in a 37°C waterbath. Transfer cells to 10 mL RPMI 1640 media in a 15 mL conical tube.
2. Centrifuge 700 rpm for 5 minutes at room temperature.
3. Resuspend cell pellet in 10 mL RPMI 1640 media and transfer to a 10-cm dish.
4. Grow LNCaP cells until about 70-80% confluency (approximately 5 X 10^6 cells).
5. Wash with warm PBS and trypsinize to detach cells.
6. Collect cells with 10 mL RPMI media and plate the cells at a high density. To maintain the cells, plate about 1 X 10^6 cells per 10-cm dish (i.e., 2 mL of the resuspension plus 8 mL RPMI). To achieve a large number of cells (i.e., 1 X 10^8 cells), plate 5 mL of this resuspension plus 15 mL RPMI media onto 2 separate 15-cm dishes. When the cells have grown to 80% confluency, trypsinize and plate onto 2 500-cm dishes. Starting from the initial thaw, growing 10^8 LNCaP cells may take at least 2-3 weeks.

LNCaP (ATCC, Cat# CRL-1740)

Farnham Lab (150720 version)

Regular culture:

1. Thaw the vial containing 1mL of LNCaP cells in a 37°C waterbath. Transfer cells to a 10cm dish containing 9mL of growth medium (RPMI 1640 with 10% FBS and optional addition of penicillin (100 units/mL), streptomycin (100 µg/mL)).
2. Incubate in 5% CO_2 incubator at 37°C overnight.
3. Discard medium carefully, add 10mL of fresh growth medium.
4. Grow cells until about 80% (+/-10%) confluence.
5. Wash cells carefully with warm PBS.
6. Trypsinize cells with 3mL of 0.05% trypsin and 0.02% EDTA at 37°C for 5 min.
7. Neutralize trypsin with 7mL of fresh growth medium.
8. Spin down cells at 470rcf for 5 min at room temperature.
9. Discard supernatant, resuspend cells with 5mL of growth medium, transfer 5mL of cells to a 15cm dish containing 15mL of growth medium growth as new passage.
10. When cells grow to about 80% (+/-10%) confluence, harvest cells by using above steps 5 to 8.
11. Freeze cell stocks by using above steps 5 to 6 with 2x volumes when cells are in a 15cm dish. Discard supernatant, resuspend cells with 3mL of freeze medium (RPMI 1640 with 20% FBS and 10% DMSO), separate into 3 cryo vials, put in foam storage box in -80°C overnight, then remove to liquid nitrogen.

DHT Treatment:

1. Grow cells as regular culture until about 80% (+/-10%) confluence.
2. Wash cells carefully with warm PBS.
3. Trypsinize cells with 3mL of 0.05% trypsin and 0.02% EDTA at 37°C for 5 min.
4. Neutralize trypsin with 7mL of fresh growth medium.
5. Spin down cells at 470rcf for 5 min at room temperature.
6. Discard supernatant, resuspend cells 20mL of phenol red free RPMI 1640 with 10% of charcoal stripped FBS, transfer cells to a 15cm dish.
7. Incubate in 5% CO2 incubator at 37°C for 2 days.
8. After the 2 days, discard medium carefully, add 20mL of fresh phenol red free RPMI 1640 with 10% of charcoal stripped FBS + 10nM DHT or equal volume of ethanol (final ethanol concentration not to exceed 0.01%). Mix DHT/ethanol thoroughly in a 50-mL blue-cap tube before adding to cells.
9. Incubate in 5% CO2 incubator at 37°C for designed time.

22Rv1 (ATCC, Cat#CRL-2505)
Farnham Lab (150923 version)

Complete growth medium: RPMI1640+10%FBS with optional addition of penicillin (100units/mL), streptomycin (100μg/mL).

Subculturing:
For cells in 15cm dish:

1. Remove and discard culture medium.
2. Briefly rinse the cell layer with PBS to remove all traces of serum that contains trypsin inhibitor.
3. Add 6-8 mL of 0.25% (w/v) Trypsin-0.53mM EDTA solution to rinse cell.
4. Discard Trypsin-EDTA solution and observe cells under an inverted microscope until cell layer is dispersed (usually within 5 to 15 minutes). **Note**: To avoid clumping do not agitate the cells by hitting or shaking the flask while waiting for the cells to detach. Cells that are difficult to detach may be placed at 37°C to facilitate dispersal.
5. Add 6-8mL of complete growth medium and aspirate cells by gently pipetting.
6. Add appropriate aliquots of the cell suspension to new culture vessels, a 1:3 to 1:6 ratio is recommended.
7. Incubate cultures at 37°C and change medium every 2-3 days.

Freeze medium: Complete growth medium supplemented with 5% (v/v) DMSO

22Rv1 (ATCC, Cat#CRL-2505)
Farnham Lab (150923 version)

Complete growth medium: RPMI1640+10%FBS with optional addition of penicillin (100units/mL), streptomycin (100µg/mL).

Subculturing:
For cells in 15cm dish:
1. Remove and discard culture medium.
2. Briefly rinse the cell layer with PBS to remove all traces of serum that contains trypsin inhibitor.
3. Add 6-8 mL of 0.25% (w/v) Trypsin-0.53mM EDTA solution to rinse cell.
4. Discard Trypsin-EDTA solution and observe cells under an inverted microscope until cell layer is dispersed (usually within 5 to 15 minutes). **Note**: To avoid clumping do not agitate the cells by hitting or shaking the flask while waiting for the cells to detach. Cells that are difficult to detach may be placed at 37°C to facilitate dispersal.
5. Add 6-8mL of complete growth medium and aspirate cells by gently pipetting.
6. Add appropriate aliquots of the cell suspension to new culture vessels, a 1:3 to 1:6 ratio is recommended.
7. Incubate cultures at 37°C and change medium every 2-3 days.

Freeze medium: Complete growth medium supplemented with 5% (v/v) DMSO

DHT treatment:
In 15cm dish:
1. After trpsinization, neutralize and plate cells in phenol red free RPMI1640+10% charcoal stripped FBS (pen/strep optional).
2. Incubate at 37°C for 48hrs until cells reach desired confluence.
3. Add 100µM DHT at ratio 1:10,000 (final conc. 10nM) to phenol red free RPMI1640+10% charcoal stripped FBS in tube.
4. Add equal volume vehicle (final conc. <0.01%) to phenol red free RPMI1640+10% charcoal stripped FBS in another tube as control.
5. Discard medium in plate carefully, add 20mL pre-mixed medium, respectively to both, test and control plates.
6. Incubate at 37°C for 4 hours and then harvest cells.

RWPE-1 (ATCC, Cat# CRL-11609)
Farnham Lab (150923 version)

Complete growth medium
The base medium for this cell line is provided by Invitrogen (GIBCO) as part of a kit: Keratinocyte Serum Free Medium (K-SFM), Kit Catalog Number 17005-042. This kit is supplied with each of the two additives required to grow this cell line:

1) 0.05mg/ml BPE - provided with the K-SFM kit
2) 5ng/mL EGF - provided with the K-SFM kit. NOTE: Do not filter complete medium.

To make the complete growth medium, you will need to add the following components to the base medium. Optional: Addition of penicillin (100units/mL), streptomycin (100µg/mL).

Thaw cells
1. Thaw the vial containing 1mL of RWPE-1 cells in a 37°C water bath. Transfer cells to a 10cm dish containing 9mL of growth medium.
2. Incubate in 5% CO^2 incubator at 37°C overnight.
3. Discard medium carefully, add 10mL of fresh growth medium.

Subculturing
For cell growth in 15cm dish:
1. Grow cells until about 80% (+/-10%) confluence.
2. Remove and discard culture medium.
3. Wash cells carefully with warm PBS.
4. Trypsinize cells with 6-8ml of 0.05% trypsin and 0.53mM EDTA at 37^oC for 5-8min.
   Note: To avoid clumping do not agitate the cells by hitting or shaking the flask while waiting for the cells to detach.
5. Neutralize trypsin with 12-14ml of PBS+2%FBS.
6. Transfer cell suspension to centrifuge tube and spin down cells at 125rcf for 5min at room temperature.
7. Discard supernatant, re-suspend cells with growth medium (K-SFM) and passage at 1:3 to 1:5 ratios (An inoculum of 2×10^4 to 4×10^4viable cells/cm^2 is recommended).
8. Incubate cultures at 37°C. Renew medium every 2 days. We recommend that you maintain cultures at a cell concentration between 4×10^4 and 7×10^4 cells/cm^2.

Cells grown under serum-free or reduced serum conditions may not attach strongly during the 24 hours after subculture and should be disturbed as little as possible during that period.

Freeze medium: Complete growth medium supplemented with 10% (v/v) DMSO and 15% FBS.

RWPE-2 (ATCC, Cat# CRL-11610)
Farnham Lab (150923 version)

Complete growth medium
The base medium for this cell line is provided by Invitrogen (GIBCO) as part of a kit: Keratinocyte Serum Free Medium (K-SFM), Kit Catalog Number 17005-042. This kit is supplied with each of the two additives required to grow this cell line:

1) 0.05mg/ml BPE - provided with the K-SFM kit
2) 5ng/mL EGF - provided with the K-SFM kit. NOTE: Do not filter complete medium.

To make the complete growth medium, you will need to add the following components to the base medium. Optional: Addition of penicillin (100units/mL), streptomycin (100µg/mL).

Thaw cells

1. Thaw the vial containing 1mL of RWPE-2 cells in a 37°C water bath. Transfer cells to a 10cm dish containing 9mL of growth medium.
2. Incubate in 5% CO^2 incubator at 37°C overnight.
3. Discard medium carefully, add 10mL of fresh growth medium.

Subculturing
For cell growth in 15cm dish:

1. Grow cells until about 80% (+/-10%) confluence.
2. Remove and discard culture medium.
3. Wash cells carefully with warm PBS.
4. Trypsinize cells with 6-8ml of 0.05% trypsin and 0.53mM EDTA at 37°C for 5-8min.
   Note: To avoid clumping do not agitate the cells by hitting or shaking the flask while waiting for the cells to detach.
5. Neutralize trypsin with 12-14ml of PBS+2%FBS.
6. Transfer cell suspension to centrifuge tube and spin down cells at 125rcf for 5min at room temperature.
7. Discard supernatant, re-suspend cells with growth medium (K-SFM) and passage at 1:3 to 1:5 ratios (An inoculum of 2×10^4 to 4×10^4viable cells/cm^2 is recommended).
8. Incubate cultures at 37°C. Renew medium every 2 days. We recommend that you maintain cultures at a cell concentration between 4×10^4 and 7×10^4 cells/cm^2.

Cells grown under serum-free or reduced serum conditions may not attach strongly during the 24 hours after subculture and should be disturbed as little as possible during that period.

Freeze medium: Complete growth medium supplemented with 10% (v/v) DMSO.

PrEC (Lonza, Cat# CC-2555)
Farnham Lab (151016 version)

Growth Medium

1. **PrEGM™ BulletKit™ Kit (CC-3166)** contains:
   a. PrEBM™ (CC-3165) Prostate Epithelial Basal Medium 500ml (no growth factors) (store at 4-8°C).
   b. PrEGM™SingleQuots™ (CC-4177) Supplements and growth factors (BPE, hydrocortisone, hEGF, epinephrine, insulin, triiodothyronine, transferrin, gentamicin/amphotericin-B and retinoic acid) (avoid refreeze by storing at -20°C upon arrival or store at 4°C for 72 hours or less and add to basal medium).
2. **ReagentPack™ (CC-5034)** contains:
   a. Trypsin/EDTA (CC-5012)
   b. Trypsin Neutralizing Solution (CC-5002)
   c. HEPES Buffered Saline Solution (CC-5022)

Subculture reagents may thaw during transport. They may be refrozen once. Store at 4°C If you plan to use within 3 days. Trypsin/EDTA solution has a limited shelf life/activation at 4°C. Immediately aliquot and refreeze at –20°C if trypsin/EDTA is thawed upon arrival. We recommend that the HEPES-BSS and the Trypsin Neutralizing Solution be stored at 4°C for no more than 1 month.

Cell Culture

1. **Preparation**
   a. Decontaminate the external surfaces of all supplement vials and the medium bottle with ethanol or isopropanol.
   b. Aseptically open each supplement vial and add the entire amount to the basal medium with a pipette.
   c. Rinse each cryovial with the medium. It may not be possible to recover the entire volume listed for each cryovial. Small losses, even up to 10%, should not affect the cell growth characteristics of the supplemented medium.
   d. Transfer the label provided with each kit to the basal medium bottle being supplemented. Use it to record the date and amount of each supplement added. We recommend that you place the completed label over the basal medium label (avoid covering the basal medium lot # and expiration date) to avoid confusion or possible double supplementation.

e. Record the new expiration date on the label based on the shelf life. Use within 1 month after SingleQuots™ are added to basal medium. Do not re-freeze.

2. **Seeding:**
   a. The recommended seeding density for PrEC is 2,500 cells/cm^2.
   b. Calculate the number of vessels to set up cultures needed based on the recommended seeding density and the surface area of the vessels being used. Do not seed cells into a well plate directly out of cryopreservation. Add the appropriate amount of medium to the vessels (1mL/5cm^2) and allow the vessels to equilibrate in 37°C and 5% CO^2 humidified incubator for at least 30 minutes.
   c. Wipe cryovial with ethanol or isopropanol before opening. Briefly twist the cap a quarter turn in a sterile field to relieve pressure and then retighten. Quickly thaw the cryovial in a 37°C water bath while being careful not to submerge the entire vial. Watch the cryovial closely and when the last sliver of ice melts, then remove it. Thawing the cells for longer than 2 minutes results in less than optimal results.
   d. Using a pipette, resuspend the cells in the cryovial and dispense cells into the culture vessels set up earlier. Gently rock the culture vessel to evenly distribute the cells and return to the incubator.
   e. Centrifugation should not be performed to remove cells from cryoprotectant cocktail. This action is more damaging than the effects of DMSO residue in the culture.
3. **Preparation for subculturing the first flask:** The following instructions are for a 25cm^2 flask. Adjust all volumes accordingly for other size flasks.
   a. Subculture the cells when they are 60%-80% confluent and contain many mitotic figures throughout the flask.
   b. For each 25cm^2 of cells to be subcultured:
      i. Thaw 2mL of Trypsin/EDTA and allow to come to room temperature.
      ii. Allow 7-10mL of HEPES Buffered Saline Solution (HEPES-BSS) to come to room temperature.
      iii. Allow 4mL of Trypsin Neutralizing Solution (TNS) to come to room temperature.
   c. Remove growth medium from 4°C storage and start warming to room temperature.
   d. Prepare new culture vessels.
   e. Subculture one flask at a time. All flasks following the first flask will be subcultured following an optimization of this protocol based on calculated cell count, cell viability, and seeding density.
4. **Subculture in sterile hood**
   a. Aspirate the medium from one culture vessel.
   b. Rinse the cells with 5mL of room temperature HEPES-BSS. DO NOT forget this step. The medium contains complex proteins and calcium that neutralize the trypsin.
   c. Aspirate the HEPES-BSS from the flask.

d. Cover the cells with 2mL of trypsin/EDTA solution.
e. Examine the cell layer microscopically.
f. Allow the trypsinization to continue until approximately 90% of the cells are rounded up. This entire process takes about 2-6 minutes, depending on cell type.
g. At this point, rap the flask against the palm of your hand to release the majority of cells from the culture surface. If only a few cells detach, you may not have let them trypsinize long enough. Wait 30 seconds and rap again. If cells still do not detach, wait and rap every 30 seconds thereafter.
h. After cells are released, neutralize the trypsin in the flask with 4mL of room temperature TNS. If the majority of cells do not detach within 7 minutes, the trypsin is either not warm enough or not active enough to release the cells. Harvest the culture vessel as described above, and either re-trypsinize with fresh, warm trypsin/EDTA solution or rinse with TNS and then add fresh, warm medium to the culture vessel and return to an incubator until fresh trypsinization reagents are available.
i. Quickly transfer the detached cells to a sterile 15mL centrifuge tube.
j. Rinse the flask with a final 2mL of HEPES-BSS to collect residual cells, and add this rinse to the centrifuge tube.
k. Examine the harvested flask under the microscope to make sure the harvest was successful by looking at the number of cells left behind. This should be less than 5%.
l. Centrifuge the harvested cells at 220xg for 5 minutes to pellet the cells.
    i. Aspirate most of the supernatant, except for 100-200µl.
    ii. Flick the cryovial with your finger to loosen the pellet.
m. Dilute the cells in 2-3mL of growth medium and note the total volume of the diluted cell suspension.
n. Determine cell count and viability using a hemacytometer and trypan blue. Make a note of your cell yield for later use.
o. If necessary, dilute the suspension with the HEPES-BSS to achieve the desired "cells/ml" and re-count the cells.
p. Use the following equation to determine the total number of viable cells.

    Total # of Viable Cells = Total cell count × percent viability/100

q. Determine the total number of flasks to inoculate by using the following equation. The number of flasks needed depends upon cell yield and seeding density. If seeding into well plates at this time, the recommended density is 10,000 cells/cm^2.

    Total # of Flasks to inoculate = Total # of viable cells/ Growth area ×Rec.SeedingDensity

r. Use the following equation to calculate the volume of cell suspension to seed into your flasks.

    Seeding Volume = Total volume of diluted cell suspension/ # of flasks as determined in previous step

s. Prepare flasks by labeling each flask with the passage number, strain number, cell type and date.
t. Carefully transfer growth medium to new culture vessels by adding 1mL growth medium for every 5cm^2 surface area of the flask (1mL/5cm^2).

u. After mixing the diluted cells with a 5mL pipet to ensure a uniform suspension, dispense the calculated volume into the prepared subculture flasks.
v. If not using vented caps, loosen caps of flasks. Place the new culture vessels into a 37°C humidified incubator with 5% CO_2.

5. **Maintenance**
   a. Change the growth medium the day after seeding and every other day thereafter. As the cells become more confluent, increase the volume of media as follows:
      i. Under 25% confluence then feed cells 1mL per $5cm^2$
      ii. 25-45% confluence then feed cells 1.5mL per $5cm^2$
      iii. Over 45% confluence then feed cells 2mL per $5cm^2$
   b. Warm an appropriate amount of medium to 37°C in a sterile container. Remove the medium and replace it with the warmed, fresh medium and return the flask to the incubator.
   c. Avoid repeated warming and cooling of the medium if the entire contents are not needed.
6. **Product warranty**
   a. CULTURES HAVE A FINITE LIFESPAN IN VITRO. Lonza warrants its cells in the following manner only if Clonetics™ media and reagents are used.
   b. Clonetics™ PrEC cryopreserved cultures are assured for experimental use for 15 population doublings.
   c. Clonetics™ PrEC proliferating cultures are assured for experimental use for 10 population doublings.
   d. Additional population doublings and subcultures are possible, but growth rate, biological responsiveness and function deteriorate with subsequent passage.
   e. PrEC can become irreversibly contact-inhibited if allowed to reach confluence. To avoid the loss of your cells and forfeiture of your warranty, subculture cells before they reach 80% confluence.
